# Thrombospondin Related Anonymous Protein Superfamily in Vector-Borne Apicomplexans: The Parasite’s Toolkit for Cell Invasion

**DOI:** 10.3389/fcimb.2022.831592

**Published:** 2022-04-06

**Authors:** Martina Soledad Paoletta, Silvina Elizabeth Wilkowsky

**Affiliations:** Instituto de Agrobiotecnología y Biología Molecular (IABIMO), Instituto Nacional de Tecnología Agropecuaria (INTA) - Consejo Nacional de Investigaciones Científicas y Técnicas (CONICET), Hurlingham, Argentina

**Keywords:** thrombospondin-related anonymous protein, TRAP family, TRP family, *Plasmodium*, *Babesia*, adhesive domains, thrombospondin type 1 repeat, von Willebrand factor type A

## Abstract

Apicomplexan parasites transmitted by vectors, including *Babesia* spp. and *Plasmodium* spp., cause severe disease in both humans and animals. These parasites have a complex life cycle during which they migrate, invade, and replicate in contrasting hosts such as the mammal and the invertebrate vector. The interaction of parasites with the host cell is mediated by adhesive proteins which play a key role in the different cellular processes regarding successful progression of the life cycle. Thrombospondin related anonymous protein (TRAP) is a superfamily of adhesins that are involved in motility, invasion and egress of the parasite. These proteins are stored and released from apical organelles and have either one or two types of adhesive domains, namely thrombospondin type 1 repeat and von Willebrand factor type A, that upon secretion are located in the extracellular portion of the molecule. Proteins from the TRAP superfamily have been intensively studied in *Plasmodium* species and to a lesser extent in *Babesia* spp., where they have proven to be functionally relevant throughout the entire parasite’s journey both in the arthropod vector and in the mammalian host. In recent years new findings provided answers to the role of TRAP proteins and in some cases the function of these adhesins during the parasite’s life cycle was redefined. In this review we will discuss the current knowledge of the diverse roles of the TRAP superfamily in vector-borne parasites from Class Aconoidasida. We will focus on the varied approaches that allowed the understanding of protein function and the relevance of TRAP- superfamily throughout the entire parasite’s cell cycle.

## Introduction

The phylum Apicomplexa comprises obligate intracellular parasites of animals including species of relevance in veterinary and public health. In particular, the class Aconoidasida includes those species transmitted by bloodsucking invertebrate vectors and contains two main orders: Piroplasmida and Haemosporida (Arisue and Hashimoto, 2015). In terms of human health, the most significant genus in Haemosporida is *Plasmodium*, which causes malaria infection leading to over 627.000 deaths a year (World Health Organization, 2021). The order Piroplasmida includes the genera *Babesia* and *Theileria* that cause disease in production and companion animals being responsible for significant economic losses worldwide (Bock et al., 2004). The major economic impact of *Babesia* is on the cattle industry and *B. bovis*, *B. bigemina* and *B. divergens* are among the most relevant species. *Babesia* infection in humans was relatively uncommon many years ago but recently there has been an increase in reports of infections by *B. microti* and *B. divergens* in immunocompromised patients, often causing death (Krause, 2019).

**Figure 1:**
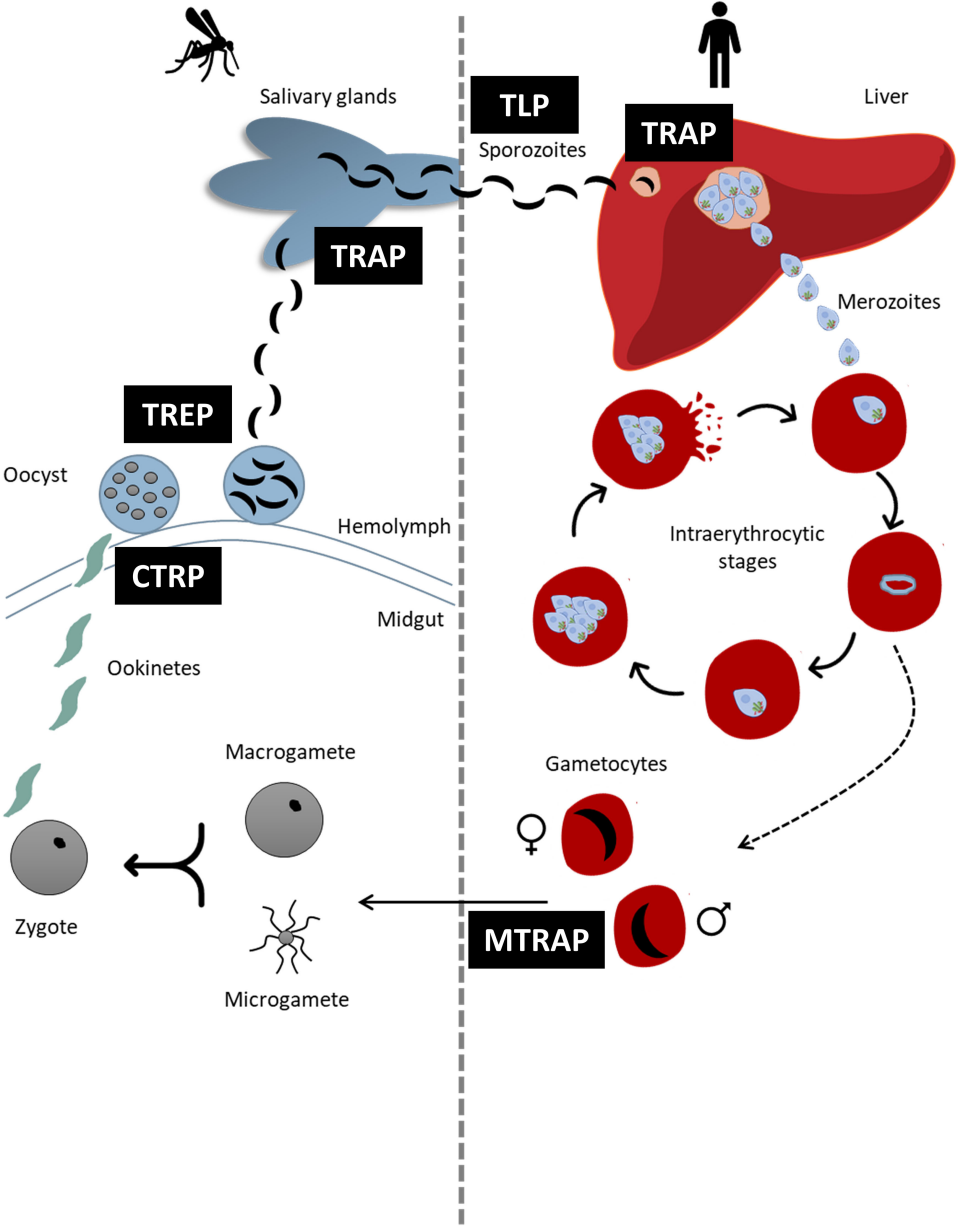
Schematic representation of the life cycle of *Plasmodium* spp. highlighting the moments in which each TRAP- family protein intervene. Each TRAP- family protein is indicated in black boxes.

All species from Aconoidasida have a complex life cycle alternating between an asexual phase in the vertebrate host and a sexual phase in the invertebrate vector (**Figure 1**) (reviewed by Jalovecka et al., 2019; Venugopal et al., 2020). In general terms, after sexual replication in the vector (ticks in Piroplasmida and mosquitoes in Haemosporida), infective sporozoites present in the salivary gland of the vector are inoculated into the host during a blood meal. Once inside the host, sporozoites infect liver cells (*Plasmodium*) or lymphocytes and endothelial cells (*Theileria*). Parasites mature and merozoites are released to invade new erythrocytes, initiating the asexual multiplication cycle. In the case of *Babesia* species, a pre-erythrocytic cycle is absent and sporozoites immediately invade erythrocytes initiating the asexual phase (Jalovecka et al., 2018). It is worth mentioning that despite having the same names, some stages of parasites from Piroplasmida and Haemosporida should not be considered identical and might express different sets of proteins interacting with the host cells.

A distinguishable characteristic of all apicomplexans is a set of apical specialized secretory organelles named rhoptries, micronemes and dense granules. These organelles store different proteins that play major roles mediating motility, invasion, and egress from host cells (Cowman et al., 2017; Arredondo et al., 2021). Motility in apicomplexans relies on a unique substrate-dependent process called gliding that allows parasites to actively move and penetrate the host cell. This particular type of movement is powered by a macromolecular complex called “glideosome” which is composed of an actomyosin system anchored in the inner membrane complex of the parasite (Soldati-Favre, 2008; Heintzelman, 2015; Frénal et al., 2017). To generate the locomotive force that propels the parasite, the glideosome interacts with host cell receptors through adhesive proteins that are released from the organelles of the apical complex and inserted into the parasite plasma membrane. These adhesin-receptor complexes are translocated backwards generating a forward movement (Keeley and Soldati, 2004; Asada et al., 2012; Frénal et al., 2017). This interaction between the parasite and the host cell is critical for the progression of infection, therefore adhesins have been the subject of numerous studies aiming at using them as vaccine candidates or drug targets to block invasion (reviewed by Morahan et al., 2009; Boucher and Bosch, 2015; Heintzelman, 2015; Frénal et al., 2017). Whereas the glideosome machinery is quite conserved across the phylum Apicomplexa, adhesins are species- and stage-specific, and expression of a broad range of these proteins allows the parasite to invade a wide variety of host cells (reviewed in Morahan et al., 2009).

Initial studies in *Plasmodium* revealed that a protein with adhesive properties, named thrombospondin related anonymous protein (TRAP), plays an essential role in gliding during sporozoite cell invasion (Robson et al., 1988; Sultan et al., 1997). Further on, other TRAP-like proteins were linked to motility and invasion of other stages of *Plasmodium* (Trottein et al., 1995; Kaiser et al., 2004; Baum et al., 2006; Heiss et al., 2008; Moreira et al., 2008).

The first TRAP protein in Piroplasmida was identified in 2004 by Gaffar et al., who discovered and characterized a neutralization sensitive protein at the apical end of *B. bovis* merozoites that resembled the architecture of *Plasmodium* TRAP and was directly involved in the recognition and invasion processes.

Many other proteins that share structural and functional characteristics with TRAP were identified in Apicomplexa, and based on the presence of some specific structural features, the TRAP superfamily was divided into two groups, the TRAP- family and the TRAP-related protein (TRP) family (Morahan et al., 2009).

In this review we will recapitulate the current knowledge and latest findings on members of the TRAP- and TRP- protein families in vector-borne parasites from Class Aconoidasida. We make a special emphasis on the reverse genetics experiments that lead to the discovery of protein function and on how novel findings changed our conception of the role of these proteins.

## TRAP- and TRP- Protein Families: Classification and Domain Architecture

Members from the TRAP- and TRP- families are integral membrane proteins with one or more adhesive domains in the extracellular portion of the molecule. The adhesive domains that can be found in TRAP- superfamily are thrombospondin type 1 repeat (TSR) and/or von Willebrand factor type A (vWA) (Morahan et al., 2009).

**Figure 2:**
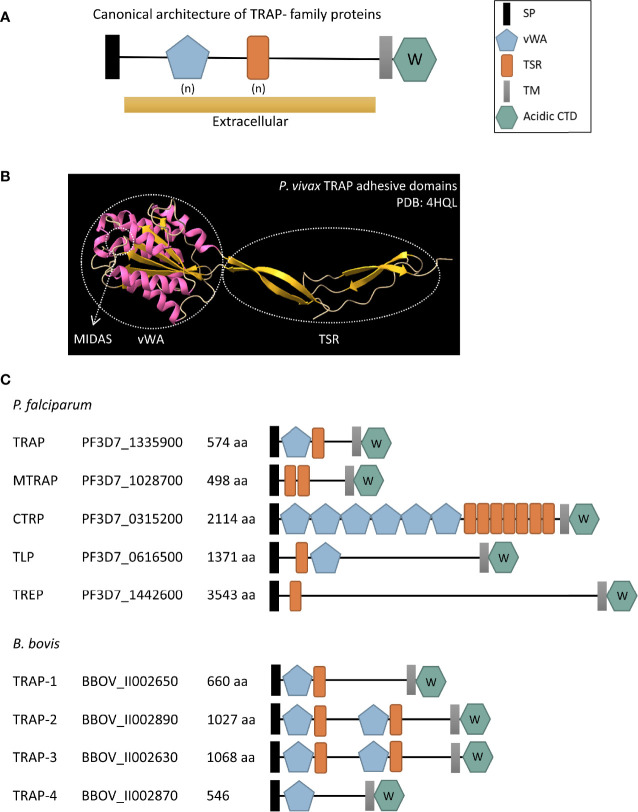
**(A)** Schematic representation of the canonical architecture of TRAP- family proteins. **(B)** Cartoon diagram of the crystal structure of the adhesive domains of *Plasmodium vivax* TRAP protein (PDB: 4HQL). α-helixes are shown in pink while β-strands are shown in yellow. The vWA and TSR domains are indicated in circles and an arrow shows the MIDAS site. **(C)** Schematic representation of TRAP- family proteins in *P. falciparum* and *B. bovis*, two relevant species from Haemosporida and Piroplasmida, respectively. SP, signal peptide; vWA, von Willebrand factor type A domain; TSR, thrombospondin type 1 repeat domain; TM, transmembrane domain; acidic CTD, acidic cytoplasmatic tail domain; aa, number of amino acids in the protein. (n) denotes a variable number of vWA and TSR extracellular domains. The W inside the acidic CTD hexagon indicates that there is a subterminal W residue within it. The accession number provided corresponds to the VEupathDB database (https://veupathdb.org/veupathdb/app).

TRAP- family proteins are stored in the micronemes of the parasites prior to secretion and have a canonical architecture of different domains (**Figure 2A**). This includes an initial signal peptide that directs them towards the secretion pathway, a variable number of vWA and/or TSR adhesive domains that are exposed to allow binding of ligands after the protein is released from the micronemes, a transmembrane domain near the C-terminal end of the protein, and a short terminal cytoplasmatic tail domain (CTD). The acidic nature of the CTD together with the presence of a sub-terminal tryptophan (W) residue within the amino acids of this domain are the hallmark of the TRAP- family and both features are decisive for classifying a protein as a member of it (Morahan et al., 2009). On the other hand, members of the TRP- family share the adhesive domains with TRAP- family proteins and are also involved in recognition and invasion of target cells, but they lack the acidic CTD and the W residue critical for TRAP function.

### TSR Domain

Thrombospondin type 1 repeat is an ancient domain that was initially identified in the glycoprotein thrombospondin I and is present in the extracellular regions of numerous proteins from a wide variety of organisms, from humans to *Drosophila* spp., *Caenorhabditis elegans* and *Plasmodium* spp. (Adams and Tucker, 2000). TSR domains mediate adhesive interactions, and the proteins that bear this domain are frequently involved in regulating matrix organization, cell–cell interactions and cell guidance (Tucker, 2004). The TSR can bind to sulphated sugar residues, particularly glycosaminoglycans (GAGs) such as heparin, heparan sulphate and chondroitin sulphate and to other extracellular matrix components (Naitza et al., 1998). The TSR is ~60 residues long with two consensus signature motifs, the N-terminal tetrapeptide WXXW (where X can be any amino acid) that can act as a heparin-binding motif and a C-terminal cluster of basic residues (Matuschewski et al., 2002; Tossavainen et al., 2006). The crystal structure of the TSR domain shows anti-parallel strands that fold into a long, thin, spiraling domain (**Figure 2B**). These strands are stabilized by stacked layers of conserved tryptophan and arginine residues between cysteine disulfide bonds (Tan et al., 2002). Based on the differences in these disulfide bonds, Tan et al. (2002) divided the TSR domains in two groups represented by the domains present in Thrombospondin-1 (Group 1) and in the related extracellular matrix protein F-spondin (Group 2). The majority of TSR containing proteins identified so far in apicomplexan parasites have a Group 2 pattern, with the exception of the TSR from *Plasmodium* PTRAMP protein that belongs to the TRP- family (see *Thrombospondin-Related Protein Family in Plasmodium* spp.) (Thompson et al., 2004).

### vWA Domain

The vWA domain was initially found in the blood glycoprotein von Willebrand factor (Sadler et al., 1985) and was then detected in an even wider range of organisms than the TSR domain, from Eukaryota (Metazoa, fungi, plants, and protists) to Eubacteria and Archaea (Whittaker and Hynes, 2002). The vWA domain is present in mammalian adhesion and cell-surface proteins including integrins, extracellular matrix, and complement components, and mediate multiple functions such as cell adhesion, migration, and signaling. This domain is ~200 amino acids long and folds in a Rossman-like tertiary structure composed of a β-sheet with both faces surrounded by α-helixes (**Figure 2B**). Its structural conformation is capable of rearranging from a closed to an open state which alters the affinity for the ligand (Schürpf and Springer, 2011). In a subgroup of vWA domains, sometimes referred as I domains, an invariant metal ion-dependent adhesion site (MIDAS) located in the middle of the ligand binding site can be found (Liddington, 2014). This motif is present in all vWA domains from apicomplexan TRAP- and TRP- family proteins identified so far.

**Figure 3:**
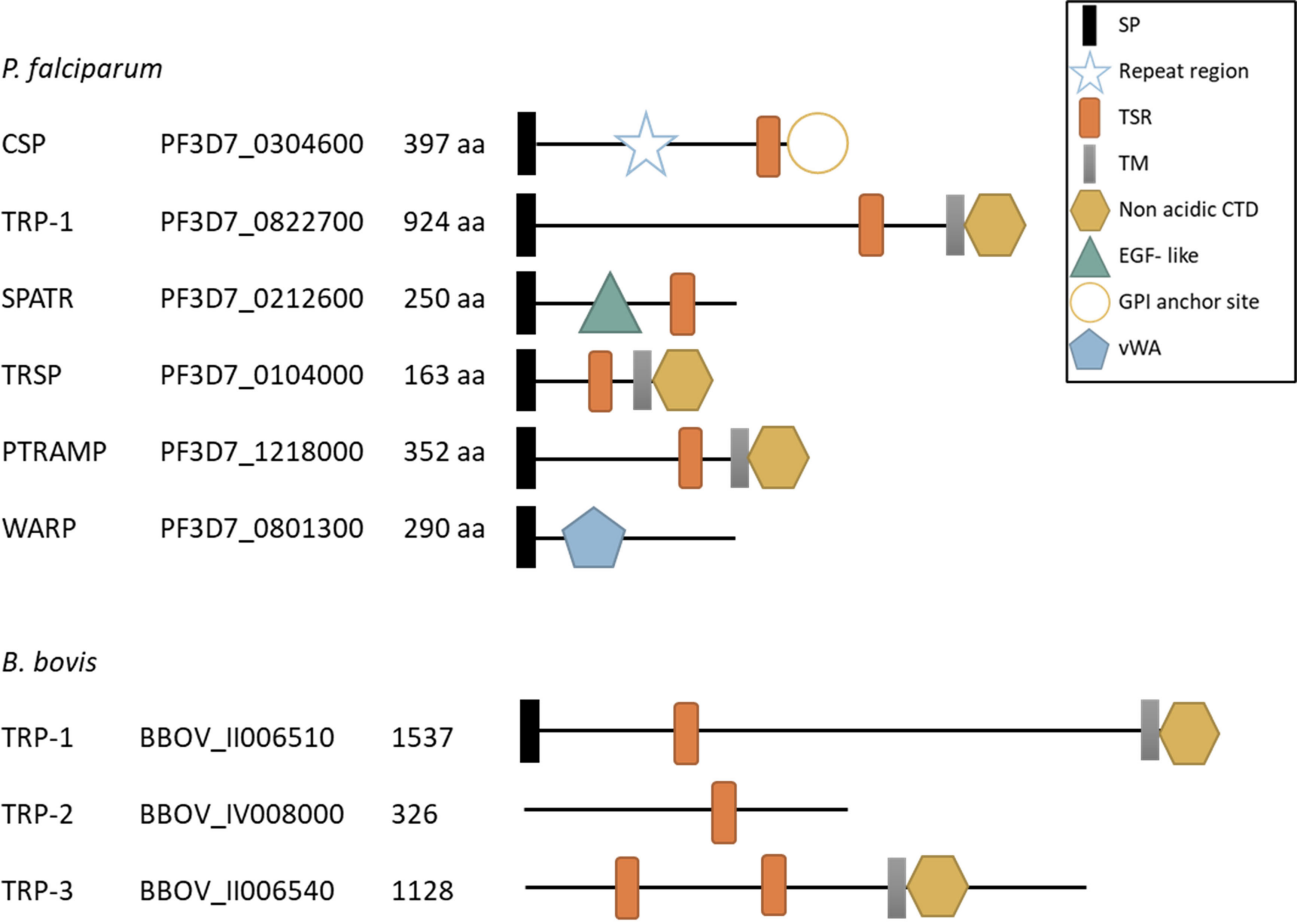
Schematic representation of TRP- family proteins in *P. falciparum* and *B. bovis*. SP, signal peptide; Repeat region, CSP central region with a variable number of repeats; TSR, thrombospondin type 1 repeat domain; TM, transmembrane domain, non acidic CTD, non acidic cytoplasmatic tail domain; EGF- like, type II EGF- like domain; GPI anchor site, glycosylphosphatidylinositol (GPI) anchor site; vWA, von Willebrand factor type A domain; aa, number of amino acids in the protein. The accession number provided corresponds to the VEupathDB database (https://veupathdb.org/veupathdb/app).

In summary, the number and position of TSR and vWA adhesive domains vary between *Plasmodium* and *Babesia* TRAP- and TRP- family proteins as shown in **Figures 2C** and **3**. Interestingly, synteny of the chromosomal regions encompassing TRAP- family genes varies both between *Plasmodium* spp. and *Babesia* spp. (**Supplementary Figure 1**). In the *Plasmodium* genus, there is a high degree of conservation of the identity and order of genes surrounding *trap*, *ctrp*, *tlp* and *trep* loci, while the chromosomal region where *mtrap* sits is highly rearranged between species. In the case of *Babesia* spp., synteny is observed in the chromosomal regions containing *trap-1* and *trap-3* genes.

As will be described in the following sections, independent lines of evidence show that when both domains are combined within other proteins, these have similar functions to TRAP, being involved in cell recognition and adhesion.

## *Plasmodium* Parasites Encode Several TRAP- Family Proteins That Play Relevant Roles Throughout the Life Cycle

*Plasmodium* parasites have a complex life cycle in which each stage migrates, invades, and replicates in different environments and encounter numerous cell types along its journey in the mammalian and invertebrate hosts (reviewed by Venugopal et al., 2020). Evidence shows that *Plasmodium* utilizes various members of the TRAP- family for motility, cell invasion, infectivity, and egress from host cells throughout the different stages of its life cycle (**Figure 1**). **Table 1** shows the stages in which *Plasmodium* TRAP- family proteins are expressed, the role they play in the different stages and the reverse genetics assays performed to elucidate their function.

**Table 1:** TRAP- family proteins in *Plasmodium*.

Protein	Stage	Location	Function in vector	Function in host	Reverse genetic assays
TRAP	Sporozoites	Microneme	Motility and invasion of salivary glands	Motility and invasion of hepatocytes	- Sultan et al. (1997): Disruption of PbTRAP results in non-gliding sporozoites with a defect for salivary gland invasion and liver infection. - Wengelnik et al. (1999): Replacement of endogenous Pb TRAP with PfTRAP results in complete complementation of PbTRAP function. Single point mutation in vWA (Asp162) or a 45 aa deletion in TSR results in poor invasion of salivary glands and impaired gliding but normal hepatocyte invasion. - Kappe et al. (1999): Point mutations in the CTD render parasites uncapable of invading mosquito salivary glands and mice liver. Mutants show an altered movement pattern. Replacing TRAP CTD with the equivalent domain of TgMIC2 has no effect on protein function. - Matuschewski et al. (2002): Point mutations in PbTRAP vWA (Thr126 or Asp157) or charge changes in conserved motifs of the TSR domain decrease sporozoite invasion of host cells but do not affect sporozoite gliding and adhesion to cells. - Ejigiri et al. (2012): Point mutations in rhomboid-cleavage site of PbTRAP results in non-motile and non-infectious parasites. - Klug et al. (2020): Complete deletion of PbTRAP vWA domain inhibits productive motility, salivary gland invasion and mice infectivity. Replacement of endogenous vWA domain with the equivalent domain of MIC2 from *T. gondii* results in less motile sporozoites still capable of invading salivary glands and mice. Point mutations in vWA that revert its charge results in parasites that normally invade salivary glands but are incapable of gliding and infecting mice. - Beyer et al. (2021): Generation of double and triple KO of PbTRAP, PbTLP and PbTREP to study interaction between adhesins revealed that they play functionally distinct and independent roles during motility and infection.
MTRAP	Merozoite/gametocyte	Microneme	ND	Egress of gametocytes from parasitophorous vacuole	- Bargieri et al. (2016): Pb and PfMTRAP KOs have a normal blood stage phenotype, but gametes are incapable of developing and egressing from the PV. - Kehrer et al. (2016): PbMTRAP KO results in a defect in gametocyte egress and oocyst formation.
CTRP	Ookinete	Microneme	Motility and invasion of mosquito midgut epithelium	ND	- Dessens et al. (1999): PbCTRP disruption results in parasites with defective motility and incapable of invading the midgut epithelium to form oocysts. - Yuda et al. (1999): PbCTRP disruption produced ookinetes that could not penetrate the midgut epithelium. - Templeton et al. (2000): PfCTRP disruption results in normal blood stage development and gametocytogenesis but impaired mosquito midgut development. - Ramakrishnan et al. (2011): Mutant PbCTRP parasites lacking all of the A domains show highly reduced motility, fail to associate with the midgut *in vivo* and are incapable of invading the midgut to form oocysts. Removal of all of the TS domains of PbCTRP render parasites fully motile and capable of developing efficiently into oocysts in mosquitoes.
TLP	Mainly in salivary gland sporozoites	Microneme	ND	Migration through tissues to reach hepatocytes	- Heiss et al. (2008): Disruption of PbTLP does not affect blood stage development or midgut and salivary gland invasion but parasites show reduced locomotion. - Moreira et al. (2008): Disruption of PfTLP and PbTLP results in parasites with normal motility, blood stage phenotype, gametocyte development and salivary gland invasion. KO parasites show a defect in migration through hepatocytes and decreased liver infectivity. - Beyer et al. (2021): *See above*
TREP	Early mosquito stages and midgut sporozoites	Microneme	Motility and invasion of salivary glands	ND	- Combe et al. (2009): PbTREP disruption show reduced salivary gland invasion and display a severe defect in gliding motility. - Steinbuechel and Matuschewski (2009): PbTREP disruption causes a gliding motility defect and an impairment in salivary gland invasion. - Beyer et al. (2021): *See above*

### TRAP in *Plasmodium* spp. Is Involved in Mosquito Salivary Gland Invasion and in the Establishment of Infection in the Mammalian Host

*Plasmodium* infection in the mammalian host initiates when an infected mosquito inoculates infective sporozoites within its saliva into the host. These highly motile sporozoites migrate through the dermis, enter the bloodstream, and infect liver cells to start intracellular replication (**Figure 1**).

The TRAP protein can be considered as the founding member of the TRAP- family and it was first identified more than 30 years ago in sporozoites from the human malaria parasite *P. falciparum* (Robson et al., 1988; Cowan et al., 1992). TRAP localizes in the micronemes and has a vWA domain with a MIDAS motif, a TSR domain (**Figure 2C**), a rhomboid cleavage motif within the transmembrane region and an Arginine-Glycine-Aspartate (RGD) cell adhesion sequence located between the TSR and transmembrane domains. Accession numbers of this and the other proteins mentioned in this review are shown in **Figure 2**.

An extensive genetic diversity was described for *P. falciparum trap* gene in clinical isolates from many endemic regions, being higher in areas with high transmission intensity. It was demonstrated that *trap* gene is under positive selection, likely due to immune system pressure since nucleotide changes are often non-synonymous leading to high antigenic diversity (Mehrizi et al., 2020).

TRAP plays a fundamental role in sporozoite motility and invasion of salivary glands in the vector as well as of vertebrate hepatocytes (**Figure 1**). The functional conservation along different *Plasmodium* species was demonstrated in experiments with the murine parasite *P. berghei*. When the endogenous TRAP was replaced with the human *P. falciparum* TRAP (PfTRAP), the wild type phenotype could be recovered throughout the entire life cycle indicating that the key domains for TRAP activity are widely conserved among the genus and they do not limit the host range infectivity (Wengelnik et al., 1999).

Many reverse genetic assays in which either the entire protein or its domains were deleted or modified allowed us to understand how TRAP performs its function (**Table 1**). For example, *P. berghei* knock out (KO) parasites lacking the complete TRAP protein were able to differentiate into sporozoites but could not reach the salivary gland. Moreover, when these KO sporozoites were injected intravenously into mice, they were unable to accomplish a productive infection (Sultan et al., 1997; Klug et al., 2020). These results evidence the importance of the dual function of TRAP in the cellular interaction with both host and vector.

The importance of the MIDAS motif of TRAP’s vWA domain was also demonstrated in experiments with *P. berghei* sporozoites. The MIDAS motif comprises five non-contiguous amino acids and as little as a single point mutation in these specific residues rendered parasites deficient in salivary gland invasion as well as in infectivity to the mammalian host, but had no consequences on sporozoite gliding (Matuschewski et al., 2002; Klug et al., 2020). Moreover, Pihlajamaa et al. (2013) showed that addition of metal chelating agents partially inhibited the binding of recombinant PfTRAP to hepatic cells, further confirming that MIDAS is relevant for parasite invasion. In a recent work done by Klug et al. (2020), the authors showed that when the complete vWA domain was removed from TRAP, the sporozoites lost their motility in a similar way as in the complete TRAP KO. Altogether these results indicate that the various biological functions of TRAP are dependent on its vWA domain and, while MIDAS is dispensable for motility, another region of the vWA domain must be implicated in this process. Surprisingly, when the vWA domain of *P. berghei* TRAP (PbTRAP) was replaced with the vWA domain of MIC2 (the TRAP ortholog in the distant apicomplexan *Toxoplasma gondii* with only a 28% of amino acidic identity), sporozoites were less motile but still invaded mosquito salivary glands (Klug et al., 2020). Furthermore, mice challenged with these MIC2 mutants became infected but with a reduced parasite burden in the liver. Additionally, amino acid mutations distal from the MIDAS site that reverted the net electrical charge of the vWA domain resulted in parasites that could normally invade salivary glands but were uncapable of gliding *in vitro* and infecting mice, suggesting that the basic charge of the vWA domain is critical for its function during transmission from the vector to the host (Klug et al., 2020).

With respect to the TSR domain, its entire deletion or the modification of the consensus sequences generated parasites that were not able to reach the salivary gland (Wengelnik et al., 1999; Matuschewski et al., 2002). Interestingly, mutant parasites in which most of the conserved portion of TSR was deleted had a severe gliding defect (Wengelnik et al., 1999). More detailed experiments by Matuschewski et al. (2002) showed that the introduction of mutations that changed the charge of the amino acids of this conserved motif had no impact on the parasite’s gliding capacity. It remains to be determined which are the key amino acids responsible for the optimal function of the TSR domain.

In sporozoites the TSR domain is glycosylated (Swearingen et al., 2016) on the CXX(S/T)C motif by the protein O-fucosyltransferase 2 (POFUT2) (Lopaticki et al., 2017). This modification is important for the stabilization and correct trafficking of TRAP to the sporozoite membrane.

The CTD of TRAP plays a fundamental role connecting the adhesive domains that extracellularly bind to the substrate, with the internal actin-myosin motor of the parasite. Modifications in the CTD residues cause alterations in parasite´s motility as well as rendering them uncapable of infecting both mosquito salivary glands and mice liver cells (Kappe et al., 1999). Additionally, it was demonstrated that TRAP CTD could be replaced by the equivalent domain of *T. gondii* MIC2, suggesting that both interact with analogous proteins (Kappe et al., 1999).

The identity of the molecule that serves as a link between adhesins and the actin-myosin motor remains controversial. For many years the consensus among researchers was that CTD attachment to actin filaments was mediated by the enzyme fructose 1,6-bisphosphate (F1,6P) aldolase (Jewett and Sibley, 2003; Bosch et al., 2007) and that locomotion resulted from the translocation of this TRAP-aldolase-actin assembly from the anterior to the posterior end of the parasite (a schematic representation can be found in Nemetski et al., 2015). Even though the CTD lacks a conserved structure, the presence of acidic amino acids as well as a conserved subterminal W residue were considered critical for this interaction and the correct performance of TRAP functions (Buscaglia et al., 2006), explaining why the classification of proteins into the TRAP- family heavily relies on the presence of this single amino acid (Morahan et al., 2009). However, more recent results performed in *T. gondii* demonstrated that the disruption of aldolase has no effect on motility or invasion when parasites grow in the absence of glucose, suggesting that the negative effects previously observed in glucose-containing media were due to a metabolic defect that resulted in the accumulation of toxic metabolites, rather than a defect in motility and invasion themselves (Shen and Sibley, 2014). Later on, a glideosome-associated connector (GAC) protein was identified in *T. gondii* and proposed as the link between adhesins and the actin filaments (Jacot et al., 2016). A GAC homologue is present in all *P. berghei* stages and adopts an apical distribution in invasive and motile stages (Jacot et al., 2016). Conclusive biochemical results about the molecular interaction of GAC with TRAP are still lacking.

After translocation to the posterior end of the parasite, TRAP molecules are cleaved by the calcium-independent rhomboid protease ROM4 (Silvie et al., 2004; Baker et al., 2006) releasing the adhesive domains that remain bound to the substrate generating the characteristic gliding trail. Mutations in the rhomboid cleavage site prevented TRAP processing and caused detrimental effects on gliding motility and cell invasion in mosquito and mammalian hosts (Ejigiri et al., 2012).

The mechanism involved in gliding mediated by TRAP is only partially understood. Studies by Münter et al. (2009) suggest that TRAP plays an essential role in the initial parasite adhesion and then coordinates the formation and turn-over of contact sites to allow continuous motility. Using traction force microscopy, they showed that the formation and rupture of these adhesion sites build up elastic energy that drives parasite motility. In line with these findings, the dynamics of formation of adhesion sites was studied by Hegge et al. (2012) demonstrating that TRAP plays a relevant role both in the initial parasite adhesion from the apical pole as well as in the formation of second and third adhesion sites necessary for productive gliding. The authors also suggest that TRAP contributes to gliding by generating traction forces in the orthogonal direction. Moreover, Song et al., 2012 showed that TRAP exists in two conformational states with different free energy and suggest that the attaching and detaching of the adhesion sites might be regulated by force-activated changes in TRAP affinity for the ligand.

As shown so far, TRAP is a multifunctional key player for gliding motility and invasion in very different host and vector environments. This fact arises the question of which are the target cell receptors to which TRAP can bind. As mentioned before, initial studies suggested that recombinant TRAP binds to sulfated GAGs and that the vWA interacts with heparin and heparan sulphate present in the hepatic cells (Müller et al., 1993; Robson et al., 1995; McCormick et al., 1999). *In vitro* studies showed that TRAP binds to human hepatocyte-derived cell lines including both heparin and non-heparin mediated interactions (Akhouri et al., 2008). Regarding the role of TRAP in the vector, Ghosh et al. (2009) demonstrated that *P. berghei* and *P. falciparum* TRAP have a high affinity binding to saglin, a specific surface protein of the mosquito salivary gland that is not modified with GAGs. Authors showed that this binding is mediated by the vWA domain and is essential for sporozoite invasion of the salivary gland.

Recently, Dundas et al. (2018) identified the human integrin αvβ3 as a host receptor for PfTRAP through protein interaction assays. The authors conclude that the interaction is mediated by both the vWA domain, including the MIDAS site, and the RGD motif and state that the TRAP-integrin interaction has a minor role in hepatocyte invasion, contrasting the aforementioned studies. The authors propose that integrin acts as an indicator for the parasite to stop gliding motility and switch to cell traversal or invasion behaviors. This conclusion also contradicts the assumption made by Klug et al. (2020) who state that TRAP acts as a poly-specific receptor providing traction for sporozoite migration and that it does not determine tissue tropism. Therefore, further studies should be done to clarify if there is a single or multiple ligands of TRAP and the exact role of the protein-ligand interaction since many questions remain to be answered towards the elucidation of its function.

### Merozoite TRAP (MTRAP), Responsible for Merozoite Invasion or Involved in Gametocyte Egress?

After sporozoites infect liver cells, they replicate and maturate into schizonts. Then, the hepatocyte ruptures releasing merozoites, which initiate the asexual multiplication cycle in the erythrocytes. Some merozoites differentiate into sexual gametocytes that, if ingested by mosquito, begin the sexual multiplication phase known as the sporogonic cycle (**Figure 1**).

Merozoite TRAP (MTRAP) was first described by Baum et al. (2006). The protein also localizes to the micronemes and has the capacity of interacting *in vitro* with aldolase (Baum et al., 2006). MTRAP has two TSR domains, a putative rhomboid-protease cleavage sequence and the acidic CTD with the conserved penultimate W residue which places it among the TRAP-family proteins (**Figure 2C**). The functional similarity of MTRAP to TRAP was demonstrated by Diaz et al. (2014) and Diaz et al. (2016) which showed that MTRAP CTD domain has the capacity of stimulating actin polymerization and that substitutions in the conserved W of the CTD abolish its binding to aldolase. Remarkably, MTRAP lacks the vWA domain and, unlike the majority of TRAP- family members, the TSR domain has a more compact structure (Uchime et al., 2012). The fact that MTRAP has many structural and functional similarities to TRAP made different authors assume that this protein might be implicated in merozoite invasion of erythrocytes and named it accordingly.

In this sense, the identification of the human erythrocyte surface protein Semaphorin-7A (a.k.a. CD108) as a receptor for *P. falciparum* MTRAP provided additional evidence to its role in merozoite red blood cell invasion (Bartholdson et al., 2012). However, when the authors tried to disrupt the MTRAP-Semaphorin-7A interaction, no defect on erythrocyte internalization was found. In addition to these findings, Riglar et al. (2016) performed a detailed analysis of the localization of MTRAP during erythrocyte entry and observed that MTRAP was not present at the merozoite tight junction, where actin and aldolase regularly are located.

Nonetheless, a study in which both *P. berghei* and *P. falciparum* MTRAP KO parasites were successfully generated, challenged the idea that MTRAP played an equivalent role as TRAP but in the merozoite stage (Bargieri et al., 2016). In this work, the authors demonstrate that MTRAP is not necessary for merozoite invasion but instead is crucial for the egress of gametocytes from the parasitophorous vacuole that surrounds *Plasmodium* parasites while developing inside host cells (**Figure 1**). These findings suggest that MTRAP is a key factor for transmission into mosquitoes and are consistent with the detection of MTRAP in gametocytes (Bargieri et al., 2016). These results were further supported by an independent research done by Kehrer et al. (2016) who concluded that MTRAP is redundant for erythrocyte stages but plays a key role for gamete egress. Altogether, these findings support a role of MTRAP in gametocyte egress but do not rule out a role of MTRAP related to parasite motility since it is possible that the actin-myosin motor is still involved in membrane rupture to facilitate egress. Bargieri et al. (2016) propose that MTRAP might link actin in gametes with an unknown ligand in the parasitophorous vacuole and, through motility, enable membrane disruption allowing the parasite’s exit. As in the case of TRAP, further research is needed to unequivocally determine the precise role and ligands of MTRAP in *Plasmodium*.

### The Circumsporozoite and TRAP-Related Protein (CTRP) Is Implicated in Ookinete Motility and Crossing the Mosquito Gut Epithelium

Inside the mosquito, gametes fuse generating zygotes that differentiate into motile and elongated ookinetes which invade the midgut of the mosquito and develop into oocysts. These oocysts then release sporozoites, which migrate and invade the mosquito’s salivary glands being ready for perpetuating the life cycle (**Figure 1**).

The Circumsporozoite and TRAP-Related Protein (CTRP) was first reported by Trottein et al. (1995) and plays an important role in ookinete stages where it localizes inside the micronemes (Dessens et al., 1999; Yuda et al., 1999; Templeton et al., 2000).

Strikingly, the architecture of CTRP contains six vWA and seven TSR domains, both contiguous, being the TRAP- family protein with the largest number of adhesive domains (**Figure 2C**). Four copies of the vWA domain are canonical and contain a MIDAS site (Kaneko et al., 2006) meanwhile only three out of the seven TSR have a canonical sequence (Trottein et al., 1995). CTRP has a predicted cleavage site that could be processed *in vitro* by the *P. falciparum* protease ROM4 (Baum et al., 2006), and the characteristic acidic CTD with the conserved W residue. It has been also demonstrated that CTRP can bind to aldolase (Heiss et al., 2008).

The role of CTRP was first explored through reverse genetics experiments in *P. berghei* and *P. falciparum* (Dessens et al., 1999; Yuda et al., 1999; Templeton et al., 2000). All studies demonstrated that CTRP KO parasites had a normal asexual phenotype and could generate regular numbers of gametocytes and ookinetes, but the latter were unable to invade mosquito midgut epithelium and develop into oocysts. CTRP is essential for midgut epithelium invasion (**Figure 1**) and KO parasites that showed no locomotion were impaired to reach or penetrate the target cells and, in consequence, were uncapable of transitioning from ookinete to oocyst. Klug et al. (2018) generated a CTRP KO mutant in which TRAP was expressed in ookinete stages. The authors observed that TRAP can complement the gliding defect of CTRP KO parasites, however the ookinetes were still uncapable of establishing invasion in mosquitos, highlighting that CTRP plays an additional role in this process, possibly related to the recognition of midgut specific ligands that trigger cell invasion.

Later on, the CTRP structure was dissected by removing either the six vWA or the seven TSR adhesive domains. Results revealed that when the protein lacked all the TSR domains, parasites generated normal oocysts in the mosquito but when all vWA were removed, ookinetes did not transition to oocysts (Ramakrishnan et al., 2011). Regarding the multiplicity of vWA domains, the authors hypothesize that the receptors of vWA in mosquito target cells might have a lower affinity that the corresponding receptors in the vertebrate host, so increasing the number of domains might augment attachment. Furthermore, parasites that had a CTRP without its vWA domains displayed a reduced motility while the movement of those that lacked the TSR domains was indistinguishable from the wild type (Ramakrishnan et al., 2011). These results are in agreement with the studies done in TRAP Klug et al. (2020) that showed that vWA was necessary for gliding motility. However, the apparent dispensability of TSR domains in CTRP contrasts with the important role of TRAP’s TSR domain in sporozoite invasion of salivary gland, raising the question of what evolutive forces favored the selection of this peculiar multi-TSR protein in *Plasmodium*.

### TRAP-Like Protein (TLP), an Adhesin Implicated in Cell Transversal

TRAP-like protein (TLP) was initially identified by a similarity search based on the TSR domain of TRAP (**Figure 2**) (Baum et al., 2006). TLP is structurally like TRAP having a single vWA including a MIDAS motif and a TSR domain (**Figure 2C**), together with a potential rhomboid cleavage site at the extracellular side of the transmembrane domain. TLP has a second cryptic vWA domain that is poorly conserved. The acidic CTD of TLP can also bind to aldolase *in vitro*, and once again the conserved W is essential for this binding (Heiss et al., 2008).

TLP transcription in vector and mammalian stages was assessed in two independent publications released almost simultaneously (Heiss et al., 2008; Moreira et al., 2008). Both studies agreed that *tlp* gene is mainly transcribed in salivary gland sporozoites and to a lower extent in gametocytes and midgut sporozoites. However, both reports disagree regarding the presence of TLP transcripts in merozoites.

The functional role of TLP was also investigated in both publications using reverse genetics. Both groups demonstrated that TLP is dispensable for asexual life cycle progression *in vivo*, and that the KO parasites showed normal gliding motility and were capable of infecting mosquito midgut and salivary glands. However, while Heiss et al. (2008) concluded that TLP plays a redundant role connecting the actin-myosin motor to the substrate, Moreira et al. (2008) demonstrated that TLP is involved in the parasite’s migration through tissues to finally reach the hepatocytes (**Figure 1**). During their life cycle, sporozoites that encounter a cell can proceed either invading it by forming a parasitophorous vacuole in which they reside or migrating through the cell causing a membrane rupture, a process known as cell transversal. This migration is characteristic of sporozoites prior to establishing infection in a selected hepatocyte (Mota et al., 2001; Coppi et al., 2007). Moreira et al. (2008) suggest that TLP might act linking the actin-myosin motor to specific receptors in different skin cells and endothelial barriers in the mammalian host. This hypothesis was further supported by experiments in which TLP KO parasites could glide but with less continuity than wild type sporozoites and that this defect could be rescued applying external forces that pushed parasites onto the substrate (Hegge et al., 2010). Furthermore, Hellmann et al. (2011) evaluated the migration capacity of the TLP KO sporozoites both *in vitro* using obstacle arrays and through *in vivo* imaging. The authors show that the KO parasites glided at a slower speed and underwent longer resting phases, probably due to an adhesion impairment, resulting in an inefficient dispersal. Finally, Quadt et al. (2016) studied the movement dynamics in TLP KO parasites and concluded that TLP serves as a linker between the substrate and the actin filaments and that it transduces the force from actin filaments towards the substrate. The authors also speculate that TLP might be implicated in the organization of actin filaments to generate the force needed for motility. Overall, the abovementioned evidence strongly suggests that TLP might act during migration and cell transversal by establishing and stabilizing parasite adhesion.

### TRAP-Related Protein (TREP) Is Required for Productive Gliding and Invasion of the Salivary Gland

TRAP-related protein (TREP) sometimes referred to as sporozoite-specific protein 6 (S6) or UOS3, is the fifth and last known TRAP- family member in *Plasmodium* and was identified in a sporozoite-enriched transcriptome (Kaiser et al., 2004). TREP transcripts are mostly found in early mosquito stages and midgut sporozoites but show very low transcript levels in salivary gland sporozoites. TREP is by far the largest TRAP- family protein and curiously, despite having an extensive extracellular cysteine-rich globular region, it has only one TSR domain and none vWA domain (**Figure 2C**). As the other members of the TRAP- family, TREP localizes on the micronemes and upon release it relocates to the sporozoite plasma membrane (Steinbuechel and Matuschewski, 2009).

Once again for this protein, two independent publications that used a reverse genetics approach to characterize TREP function were published simultaneously (Combe et al., 2009; Steinbuechel and Matuschewski, 2009). In this case, both studies arrived at the same conclusions: TREP is dispensable for erythrocytic stage development, gametocytogenesis, transmission to the mosquito, and oocyst formation in midguts. However, TREP KO sporozoites were drastically impaired in salivary gland invasion. When these KO parasites were injected into mice, they were able to achieve a productive infection reinforcing the idea that TREP does not play a relevant role in liver and erythrocytic stages. Both groups also demonstrated that KO parasites showed a defective gliding motility and therefore they conclude that TREP is important for sporozoite gliding and salivary gland invasion (**Figure 1**). Finally, Hegge et al. (2012) showed that TREP KO sporozoites are able to establish the first out of the three adhesion sites required for gliding but fail to establish the second and third ones which could explain the observed impairment in salivary gland invasion.

### *Plasmodium* TRAP- Family Adhesins: Are They Linked?

As shown so far, TRAP- family proteins are expressed in different stages of the parasite’s life cycle with important roles in within them. Interestingly, three out of the five TRAP- family proteins (TRAP, TLP and TREP) are expressed simultaneously during sporozoite stages (**Table 1**). This arises the question of whether there is an interaction between each other to fulfill their function, or if they all act independently.

This question was recently addressed by Beyer et al. (2021) by generating the three possible double KO combinations as well as a triple KO line. As expected, all four parasite lines showed normal blood stage development and oocyst formation, which is consistent with the fact that neither of these proteins are expressed during these stages. After analyzing the adhesion capacity, productive gliding and transmission to mice of the mutant lines, the authors conclude that all adhesins act independently since the phenotypes of the double and triple KOs were equivalent to that of the dominant gene. The authors only observed a pronounced adhesion defect in the double TRAP/TREP KO and this distinct phenotype is in accordance with the results reported by Hegge et al. (2012) indicating that both adhesins are responsible for the formation of adhesion sites needed for productive gliding. The fact that the triple KO, despite not achieving productive gliding, was still capable of performing a waving movement and patch gliding demonstrates that other proteins are involved in sporozoite adhesion to the substrate. Some of these proteins could be members of the TRAP-related protein family and will be discussed in the following section.

## Thrombospondin-Related Protein Family in *Plasmodium* spp.

The members of this second protein family, named thrombospondin-related protein (TRP) family, share some functional domains with TRAP proteins related to host-cell recognition and invasion, but they lack the characteristic acidic CTD domain with the W residue (**Figure 3**) that appears to be determinant for interaction with the actin-myosin motor.

### Circumsporozoite Protein (CSP)

The most studied member of the TRP- family is *Plasmodium* circumsporozoite protein (CSP) that is the major sporozoite surface protein and the target antigen of one the most advanced malaria vaccines, RTS,S and R21.

CSP structure contains an N-terminal domain that binds heparan sulphate proteoglycans, a protease cleavage site (region I), a central region with a variable number of repeats, a short-conserved region (region III), and a TSR domain in the C-terminal end with a string of positively charged amino acids (region II+) followed by a glycosylphosphatidylinositol (GPI) anchor site (**Figure 3**). The crystal structure of the TSR domain shows a dissimilar fold containing a hydrophobic pocket formed by highly conserved amino acids and which biological implications are yet unknown (Doud et al., 2012). Likewise to TRAP, the TSR domain of CSP is subjected to O-fucosylation by the enzyme POFUT2, however the biological implications of this modification is still unclear (Lopaticki et al., 2017).

CSP plays a critical role during vertebrate and mosquito stages. In the mosquito, CSP is involved in sporozoite formation and exit from the midgut oocysts, and invasion of salivary glands (Ménard et al., 1997; Myung et al., 2004; Wang et al., 2005). In the mammalian host, CSP is necessary for hepatocyte invasion (Cerami et al., 1994). Even though there is some evidence that suggests that CSP may influence gene expression of the infected hepatocyte providing a favorable environment for parasite development (Singh et al., 2007), a deeper characterization of this function is still necessary.

Several authors have provided evidence that suggests that CSP can adopt two different conformations (Coppi et al., 2005; Coppi et al., 2011; Herrera et al., 2015). Both studies propose a model in which the open form is present in sporozoites as they develop within the mosquito midgut and later on is processed adopting a close conformation that conceals the TSR domain. This collapsed conformation is maintained throughout the journey to the liver, and then, prior to hepatocyte invasion and during sporozoite development in the mosquito, it reverts to the open form exposing the TSR domain. Furthermore, Coppi et al. (2011) showed that when the TSR domain is prematurely exposed, sporozoites are retained in the dermis and cannot reach the liver. This model is consistent with reverse genetics assays done by Wang et al. (2005) that show that mutations in four positively charged amino acids from region II+ prevents the exit of sporozoites from oocysts and in consequence salivary gland invasion. It was also demonstrated that in the mammalian host, this region is also required for hepatocyte invasion. These results are in agreement with the work done by Tewari et al. (2002) in which they generated *P. berghei* mutants where the endogenous CSP was replaced by the *P. falciparum* counterpart, either complete or depleted from region II+. The authors showed that while both lines have a defect in salivary gland invasion, this was significantly higher in those sporozoites that lacked region II+. Also, the region II+- depleted sporozoites had a marked defect in infection of the vertebrate host.

The importance of the central repeat region was addressed by Ferguson et al. (2014) who demonstrated that when this specific part was removed from CSP, mutant parasites could still form oocysts, yet sporozoites failed to develop in a normal fashion and instead presented abnormalities such as a disorganized inner membrane complex formation and agglutination.

The RTS,S malaria vaccine includes a fragment of the central repeat domain of CSP together with the C-terminal end that contains T- and B-cell epitopes, coupled with the N-terminal end of the hepatitis B surface antigen. This vaccine aims at directing the immune system against sporozoites immediately after entering the bloodstream and therefore preventing hepatocyte invasion. The highly repetitive nature of the vaccine design also provides an enhanced presentation of the antigen to the immune system. The RTS,S vaccine has successfully provided a 18-36% protection against clinical malaria during Phase III trials (RTS,S Clinical Trials Partnership, 2015) and has recently begun a large-scale vaccination program in areas with high malaria transmission. Based on the outcome of this ongoing implementation, in October 2021 the WHO recommended the RTS,S vaccine for broad use among young children in areas with moderate to high transmission of *P. falciparum* malaria (World Health Organization, 2021). The design, immunogenicity and outcomes of the RTS,S vaccine were recently reviewed in detail (Laurens, 2020; Martins de Almeida et al., 2021).

As previously described for TRAP, there is also a high variability in *csp* alleles which makes this gene a good molecular marker that has been exploited in the study of *P. falciparum* isolates to better understand epidemiology and infection dynamics (Lerch et al., 2017). On the down side, this high degree of polymorphism threatens the efficacy of CSP-based RTS,S malaria vaccines against genotypic variants.

Recently, Noe et al. (2021) applied computational vaccinology approaches to predict conserved epitopes across reported sequence variants to help improve CSP- based malaria vaccines.

### Other TRP- Family Proteins

Several other TRP- family proteins have been characterized in *Plasmodium* spp. (**Figure 3**). An example is *Plasmodium* thrombospondin-related protein 1 (TRP-1), which is expressed by sporozoites and localizes at the outer side of their plasma membrane (Lindner et al., 2013). The protein has a single TSR domain, a TM domain and a CTD without the subterminal W residue (**Figure 3**). TRP-1 plays an important role during mosquito stages allowing the parasite to exit from the oocysts and enter mosquito hemolymph to reach and invade the salivary gland (Klug and Frischknecht, 2017).

Another member of this group is the Secreted Protein with Altered Thrombospondin Repeat (SPATR). This protein has a distorted TSR domain and a cysteine-rich motif that resembles a type II EGF- like domain, also known to be involved in adhesion and protein-protein interactions (**Figure 3**). SPATR is expressed during sporozoite, merozoite and gametocyte stages (Chattopadhyay et al., 2003; Mahajan et al., 2005) and localizes in the rhoptries of the parasite (Gupta et al., 2020). A recent reverse genetics study demonstrated that SPATR is not essential neither for parasite development in mosquito nor for liver infection but plays an essential yet unknown role during blood stages since SPATR KO parasites failed to establish blood stage infections (Gupta et al., 2020).

A fourth member is the *Plasmodium* thrombospondin-related sporozoite protein (TRSP) which contains a TSR domain and a transmembrane domain followed by a short nonacidic CTD (**Figure 3**). It is expressed in sporozoites and localizes in the rhoptries (Kaiser et al., 2004; Labaied et al., 2007). The role of the protein in *Plasmodium* lifecycle is unclear since two independent works showed contrasting results. Labaied et al. (2007) showed that depletion of TRSP caused a defect in hepatocyte invasion. However, Costa et al. (2018) demonstrated that TRSP KO parasites were able to reach and infect liver cells and suggest that discrepancies with the prior research might be due to the differences in the genetic background of the parasite strains used in both studies.

The fifth member or the TRP-family is the *Plasmodium* thrombospondin-related apical merozoite protein (PTRAMP). This protein is expressed in merozoite stages and localizes in the rhoptry bulbs (Siddiqui et al., 2013). PTRAMP has a TSR domain (the only *Plasmodium* TSR classified as a Group 1 domain), a TM domain and a short, acidic CTD that lacks the characteristic W of TRAP- family proteins (**Figure 3**). Attempts of disrupting the *ptramp* gene were unsuccessful suggesting that this is an essential gene for parasite survival (Thompson et al., 2004). Antibodies against *P. falciparum* TRAMP inhibited RBC invasion and limited parasite growth *in vitro*, supporting the idea that the protein is critical for invasion (Siddiqui et al., 2013).

A sixth TRP- family protein, named von Willebrand factor A domain-related protein (WARP) is expressed in ookinetes and localized in the micronemes as a soluble protein. This protein has a vWA domain and lacks both TSR and TM domains (**Figure 3**) therefore it is either secreted or remains attached to the membrane by interacting with other membrane attached proteins (Li et al., 2004). The generation of a WARP KO line showed that the lack of WARP had only a minor effect on the number of oocysts formed and these produced a normal number of infective sporozoites, demonstrating that this protein is not essential for development in mosquitos (Ecker et al., 2008).

## TRAP- Superfamily Proteins in *Babesia* spp. and *Theileria* spp.

The first TRAP- family protein in Piroplasmida was characterized in *Babesia bovis* in 2004 by the work of Gaffar et al., more than 15 years later than the report of the *Plasmodium* ortholog. Its later discovery, together with the fact that *Babesia* and *Theileria* parasites are less studied than *Plasmodium*, limits our current knowledge on the repertoire of Piroplasmida adhesive proteins. The successful genetic manipulation of *Babesia* and *Theileria* has been challenging and well established protocols for stable transfection in parasites of this order have become available more than 10 years later than those for *Plasmodium* and *Toxoplasma* (Suarez et al., 2017). Up to date, there are no reverse genetics studies of TRAP proteins in Piroplasmida and most of protein functions are hypothetically assigned based on orthology with *Plasmodium* and *Toxoplasma*.

However, with the advance of genome sequencing technologies, multiple genes encoding TRAP- superfamily proteins were identified in several Piroplasmida genomes. The first description was done by Montenegro et al. (2020) who performed an exhaustive search for TRAP- and TRP- family proteins in all available *Babesia* and *Theileria* genomes, identifying a variable number of protein coding sequences in the different species. The only *Babesia* spp. and *Theileria* spp. TRAP protein that has a direct ortholog in *Plasmodium* is TRAP-1. A phylogenetic analysis on all the Piroplasmida TRAP- family members demonstrated that TRAP-1 from all species cluster together, while a separate branch clusters the paralogs TRAP-2 and -3 together (Montenegro et al., 2020). Interestingly, even though the size of *P. falciparum* genome is almost two times larger than the *B. bovis* genome (22.8 Mbp vs. 8.2 Mbp, Brayton et. al, 2007) the number of TRAP- superfamily proteins is similar between both reference species (**Figures 2C** and **3**).

The architecture of TRAP proteins in Piroplasmida is similar to those of Haemosporida, combining different number of TSR and vWA domains in the extracellular portion of the protein and conserving the acidic CTD with the characteristic W residue (**Figure 2C**). Nonetheless, while in *Plasmodium* the subterminal W is invariably in the penultimate position, in *Babesia* and *Theileria* this residue occupies the fifth or sixth position from the C-terminal end (Montenegro et al., 2020).

**Figure 4:**
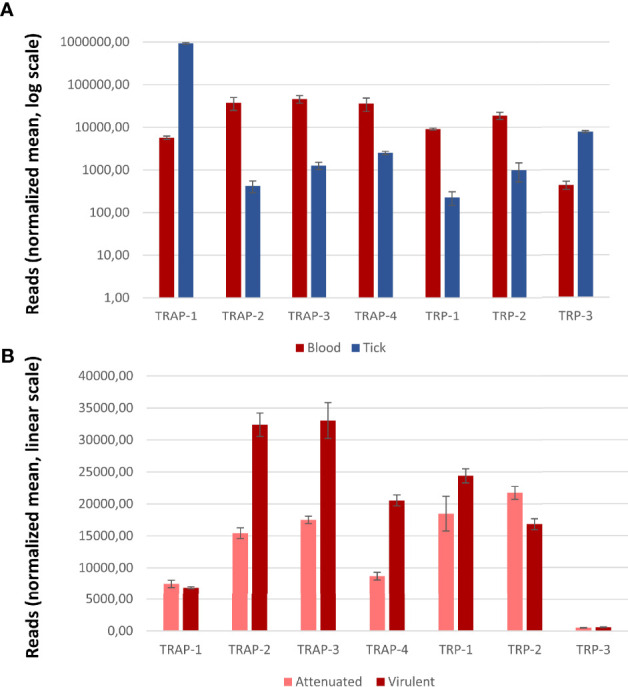
Transcription of *Babesia bovis* genes coding for TRAP- and TRP- family proteins. **(A)** Transcription patterns from blood and tick stages (normalized reads, log scale). **(B)** Transcription patterns from virulent and attenuated strains (normalized reads, linear scale). Gene transcription was considered significantly and differentially regulated if |log fold change (FC)| ≥ 1 and false detection rate (FDR) < 5%. Error bars represent S.D. Significant differences were obtained for all genes except for TRAP-1 and TRP-3.

Among Piroplasmida, *B. bovis* has four TRAP- family proteins (Terkawi et al., 2013). The analysis from two transcriptomic datasets (Pedroni et al., 2013; Ueti et al., 2020) showed that all genes are transcribed in both merozoites and kinetes (the parasitic stages located in ticks hemolymph). TRAP-1 is overexpressed in kinetes while the other three members are overexpressed in merozoites (**Figure 4**). While TRAP-1 transcription remains unaltered in blood stages of strains with contrasting virulence phenotypes, TRAP-2, -3 and -4 have higher transcription rates in blood stages of virulent strains (**Figure 4**), suggesting a relevant role in the parasite’s virulence.

In *Babesia*, TRAP-1 has been studied in *B. bovis* (Gaffar et al., 2004), *B. bigemina* (Montenegro et al., 2020) and *B. orientalis* (Yu et al., 2018) mainly upon expression of recombinant forms of the protein and *in vitro* invasion assays. Overall, results between species are consistent and show that *Babesia* TRAP-1 has a comparable modular structure to that of *Plasmodium* TRAP (**Figure 2C**), including the MIDAS site in the vWA domain and the putative rhomboid cleavage site. *B. bovis* genome encodes five homologues of rhomboid proteases that might be responsible for this cleavage (Terkawi et al., 2013), but this has not been experimentally tested so far.

TRAP-1 was detected at the apical end of the merozoites in the different *Babesia* species, consistent with a micronemal localization (Gaffar et al., 2004; Yu et al., 2018). Contrary to *Plasmodium*, *Babesia* TRAP-1 is also expressed during the erythrocytic stages and antisera raised against the recombinant protein showed a significant degree of inhibition of erythrocytic infection both for *B. bovis* and *B. bigemina*, suggesting that TRAP-1 would play an important but not essential role in red blood cell invasion, probably mediating adhesion to a membrane receptor (Gaffar et al., 2004; Montenegro et al., 2020). Both findings in *Babesia* differ from what was found for *Plasmodium* spp. (Gantt et al., 2000). It is worth mentioning that *Babesia* parasites do not undergo a liver stage prior to initiating the erythrocytic stage so the sporozoites inoculated by the tick bite directly target the erythrocyte. This difference in the life cycle in addition to different vectors and modes of transmission may explain the differential expression of TRAP orthologs in both orders. The increased transcription of TRAP-1 in kinetes suggests that this protein might be more relevant in vector stages than in blood stages, as seen for *Plasmodium* TRAP. In addition, no significant differences were found in transcription patterns (**Figure 4**) between blood stages of virulent and attenuated strains, reinforcing the hypothesis that TRAP would be more relevant in vector stages.

The other TRAP- family member studied so far in Piroplasmida is TRAP-2 (sometimes referred to as P18). *In vitro* assays using the recombinant *B. gibsoni* protein demonstrated that TRAP-2 can bind to erythrocytes (Zhou et al., 2006), and the CTD is able to bind aldolase through the subterminal W residue playing a major role (Goo et al., 2013). Yet, further experiments will be required to confirm if aldolase or a GAC ortholog are the link to the actin-myosin motor in Piroplasmida.

Studies on *B. bovis* TRAP-2 performed by Terkawi et al. (2013) show that the protein localizes in the apical end of intracellular merozoites and is then translocated to the surface of extracellular forms. Consistent observations were reported for the *B. orientalis* ortholog (Zhan et al., 2019). Furthermore, truncated forms of *B. bovis* TRAP-2 were detected in the supernatant of the culture, indicating that this protein is proteolytically processed and shed from the parasite, as seen in *Plasmodium*. Also, the same authors show that TRAP-2 is capable of binding erythrocytes and that sera raised against the recombinant protein significantly inhibit merozoite invasion of the host cell and that the reduced invasion was not due to an impairment in gliding motility. These findings are supported by the transcription data from *B. bovis* TRAP-2 that shows that the number of transcripts in blood stages is significantly higher than in tick stages, and that TRAP-2 transcripts in the virulent strain double the number of those in the attenuated counterpart (**Figure 4**). Despite not being direct evidence of the protein function, all the above-mentioned findings support the idea that TRAP-2 functions in the attachment of the merozoite prior to invasion and to a possible role in virulence.

The conserved protein domain architecture and the results described here suggest that Piroplasmida TRAP- family members are also involved in the events of invasion and motility, in a similar way as *Plasmodium* TRAP- family proteins. Still, further research is necessary to unequivocally assign function by reverse genetics or other studies and determine in which developmental stages these proteins are involved.

Regarding the TRP- family in Piroplasmida, the only evidence so far is the identification of either 2 or 3 genes in the genomes of different *Babesia* and *Theileria* species (Montenegro et al., 2020). Since there are no functional studies on the role of these proteins in Piroplasmida, insights of their relevance can only be obtained based on transcription analysis (**Figure 4**). According to this, we could hypothesize that in *B. bovis* TRP-1 might play a relevant role in blood stages from virulent strains while TRP-3 could act during tick stages.

## The TRAP-Superfamily Proteins in Other Apicomplexans

Members of the TRAP- superfamily were also identified in other parasites from the phylum Apicomplexa that are not transmitted by vectors. Interestingly, in these species the number of TRAP-superfamily proteins is reduced to one or two members, which could be related to the fact that the parasite needs to move and invade in less diverse environments.

The genome of the human pathogen *Toxoplasma gondii* codes for a single TRAP homologue named MIC2 that comprises one vWA domain, six TSR domains and the acidic CTD with the conserved W residue (Boucher and Bosch, 2015). As shown by KO assays, TgMIC2 plays an important, yet non-essential role in motility and host cell invasion, being involved in mediating the formation of attachment sites (Gras et al., 2017). Interestingly, TgMIC2 is constantly associated to TgM2AP forming a hetero-hexameric complex and disruption of TgM2AP causes an incorrect localization of TgMIC2 and an invasion defect (Huynh et al., 2003). As previously mentioned, the interaction of TgMIC2 with the actin-myosin motor is believed to be mediated by a glideosome-associated connector (GAC) (Jacot et al., 2016).

In other apicomplexan parasites of medical or veterinary importance, several TRAP- family proteins were identified but no reverse genetics assays were done to unequivocally assign their function. In the bovine parasite *Neospora caninum*, two TRAP- family proteins were identified, named NcMIC2 and NcMIC2-like1 (Lovett et al., 2000; Pereira et al., 2011). Both localize in the micronemes, and antisera raised against the adhesive domains of NcMIC2-like1 inhibited the *in vitro* invasion which suggests a role in invasion process. Avian pathogens from the *Eimeria* genus also have two TRAP family proteins, named MIC1 and MIC4, that localize in micronemes (Tomley et al., 2001) and show a differential expression during the life stages of the parasites. Finally, a bioinformatics analysis done on the human pathogen *C. parvum* genome identified a total of 12 TRAP-family proteins with TSR domains (Deng et al., 2002). Two of them, TRAP-C1 and TSP8, were characterized to some extent and demonstrated to be located in the apical end of the parasite (Spano et al., 1998; Putignani et al., 2008).

## Discussion

Vector borne apicomplexan parasites are a major constraint in terms of human and veterinary health and the development of novel drugs and more efficient vaccines is an urgent need, especially in the context of global changes in climate, use of land and closer human-animal interfaces. To improve the control strategies on vector-borne diseases, it remains indispensable to gain a deeper knowledge of fundamental processes involved in parasite´s life cycle progression and virulence, particularly the proteins involved in initial attachments, internalization, and cell transversion.

Adhesins from TRAP- and TRP- families are key players in the gliding and invasion of target cells in Aconoidasida as well as other members of Apicomplexa and, as demonstrated in this review, are functionally relevant throughout the entire parasite’s journey both in the arthropod vector and in the mammalian host. Using the same adhesive domains in different host and tissue contexts, parasites can fulfill various tasks including migration, invasion and egress from diverse epithelia and cell types. Therefore, these adhesins could be attractive targets for developing specific control measures to avoid the above-mentioned processes.

Despite the great interest of the research community in the study of TRAP- and TRP- family proteins, there are still controversies and knowledge gaps that need to be addressed to have a better understanding of their precise role in parasite’s fundamental processes. On the one hand, the definition of the ligands that interact with these proteins is critical not only for the comprehension in parasite’s biology but also to analyze if blocking such interaction is a feasible alternative for disease control. On the other hand, the role of adhesins in Piroplasmida is still in early stages and should be further studied through reverse genetic approaches. In addition, complementation studies using TRAP proteins from *Plasmodium* could be an interesting way to evaluate the conservation of protein function between Piroplasmida and Haemosporida. Even though the genetic manipulation of *Babesia* and *Theileria* has historically been a limitation for such studies, with the advent of new tools like CRISPR-Cas9 that benefits from versatility, simplicity, and low costs we can now address new questions and unequivocally assign the role of TRAP proteins in Piroplasmida.

## Author Contributions

SW contributed to conception, design, and funding acquisition for the review. MP wrote the first draft of the manuscript. Both authors worked on manuscript revision and approved the final version.

## Funding

This work was supported by the grant PICT 2018-2031 from Agencia Nacional de Promoción Científica y Tecnológica. MP is a member of Instituto Nacional de Tecnología Agropecuaria (INTA, Argentina) and SW is a career member of the Consejo Nacional de Investigaciones Científicas y Técnicas (CONICET, Argentina).

## Conflict of Interest

The authors declare that the research was conducted in the absence of any commercial or financial relationships that could be construed as a potential conflict of interest.

## Publisher’s Note

All claims expressed in this article are solely those of the authors and do not necessarily represent those of their affiliated organizations, or those of the publisher, the editors and the reviewers. Any product that may be evaluated in this article, or claim that may be made by its manufacturer, is not guaranteed or endorsed by the publisher.

## Supplementary Material

Supplementary Figure 1 Synteny of the TRAP-family loci between **(A)** the most relevant *Plasmodium* spp. or **(B)** all sequenced *Babesia* spp. The gene loci corresponding to the TRAP- family genes in the reference species *P. falciparum* or *B. bovis* are indicated with a green square. Conservation of surrounding genes is shown as shaded areas.

## References

[B1] Adams J. C. Tucker R. P. (2000). The Thrombospondin Type 1 Repeat (TSR) Superfamily: Diverse Proteins With Related Roles in Neuronal Development. Dev. Dyn. 218, 280–299. doi: 10.1002/(SICI)1097-0177(200006)218:2<280::AID-DVDY4>3.0.CO;2-0 10842357

[B2] Akhouri R. R. Sharma A. Malhotra P. Sharma A. (2008). Role of *Plasmodium falciparum* Thrombospondin-Related Anonymous Protein in Host-Cell Interactions. Malar. J. 7, 1–11. doi: 10.1186/1475-2875-7-63 18426606 PMC2373790

[B3] Arisue N. Hashimoto T. (2015). Phylogeny and Evolution of Apicoplasts and Apicomplexan Parasites. Parasitol. Int. 64, 254–259. doi: 10.1016/j.parint.2014.10.005 25451217

[B4] Arredondo S. A. Schepis A. Reynolds L. Kappe S. H. I. (2021). Secretory Organelle Function in the *Plasmodium* Sporozoite. Trends Parasitol. 37, 651–663. doi: 10.1016/j.pt.2021.01.008 33589364

[B5] Asada M. Goto Y. Yahata K. Yokoyama N. Kawai S. Inoue N.. (2012). Gliding Motility of *Babesia bovis* Merozoites Visualized by Time-Lapse Video Microscopy. PloS One 7, e35227. doi: 10.1371/journal.pone.0035227 22506073 PMC3323635

[B6] Baker R. P. Wijetilaka R. Urban S. (2006). Two *Plasmodium* Rhomboid Proteases Preferentially Cleave Different Adhesins Implicated in All Invasive Stages of Malaria. PloS Pathog. 2, 0922–0932. doi: 10.1371/journal.ppat.0020113 PMC1599764 17040128

[B7] Bargieri D. Y. Thiberge S. Tay C. L. Carey A. F. Rantz A. Hischen F.. (2016). *Plasmodium* Merozoite TRAP Family Protein is Essential for Vacuole Membrane Disruption and Gamete Egress From Erythrocytes. Cell Host Microbe 20, 618–630. doi: 10.1016/j.chom.2016.10.015 27832590 PMC5104695

[B8] Bartholdson S. J. Bustamante L. Y. Crosnier C. Johnson S. Lea S. Rayner J. C.. (2012). Semaphorin-7A is an Erythrocyte Receptor for *P. Falciparum* Merozoite-Specific TRAP Homolog, MTRAP. PloS Pathog. 8, 1003031. doi: 10.1371/journal.ppat.1003031 PMC3499583 23166499

[B9] Baum J. Richard D. Healer J. Rug M. Krnajski Z. Gilberger T. W.. (2006). A Conserved Molecular Motor Drives Cell Invasion and Gliding Motility Across Malaria Life Cycle Stages and Other Apicomplexan Parasites. J. Biol. Chem. 281, 5197–5208. doi: 10.1074/jbc.M509807200 16321976

[B10] Beyer K. Kracht S. Kehrer J. Singer M. Klug D. Frischknecht F. (2021). Limited *Plasmodium* Sporozoite Gliding Motility in the Absence of TRAP Family Adhesins. Malar. J. 20, 1–12. doi: 10.1186/s12936-021-03960-3 34717635 PMC8557484

[B11] Bock R. Jackson L. de Vos A. Jorgensen W. (2004). Babesiosis of Cattle. Parasitology 129, S247–S269. doi: 10.1017/S0031182004005190 15938514

[B12] Bosch J. Buscaglia C. A. Krumm B. Ingason B. P. Lucas R. Roach C.. (2007). Aldolase Provides an Unusual Binding Site for Thrombospondin-Related Anonymous Protein in the Invasion Machinery of the Malaria Parasite. Proc. Natl. Acad. Sci. U. S. A. 104, 7015–7020. doi: 10.1073/pnas.0605301104 17426153 PMC1855406

[B13] Boucher L. E. Bosch J. (2015). The Apicomplexan Glideosome and Adhesins - Structures and Function. J. Struct. Biol. 190, 93–114. doi: 10.1016/j.jsb.2015.02.008 25764948 PMC4417069

[B14] Brayton K. A. Lau A. O. T. Herndon D. R. Hannick L. Kappmeyer L. S. Berens S. J.. (2007). Genome Sequence of *Babesia bovis* and Comparative Analysis of Apicomplexan Hemoprotozoa. PloS Pathog. 3 (10), e148. doi: 10.1371/journal.ppat.0030148 PMC2034396 17953480

[B15] Buscaglia C. A. Penesetti D. Tao M. Nussenzweig V. (2006). Characterization of an Aldolase-Binding Site in the Wiskott-Aldrich Syndrome Protein. J. Biol. Chem. 281, 1324–1331. doi: 10.1074/jbc.M506346200 16278221

[B16] Chattopadhyay R. Rathore D. Fujioka H. Kumar S. De la Vega P. Haynes D. (2003). PfSPATR, a *Plasmodium falciparum* Protein Containing an Altered Thrombospondin Type I Repeat Domain Is Expressed at Several Stages of the Parasite Life Cycle and Is the Target of Inhibitory Antibodies. J. Biol. Chem. 278, 25977–25981. doi: 10.1074/jbc.M300865200 12716913

[B17] Cerami C. Frevert U. Sinnis P. Takacs B. Nussenzweig V. (1994). Rapid Clearance of Malaria Circumsporozoite Protein (CS) by Hepatocytes. J. Exp. Med. 179, 695–701. doi: 10.1084/jem.179.2.695 8294876 PMC2191367

[B18] Combe A. Moreira C. Ackerman S. Thiberge S. Templeton T. J. Ménard R. (2009). TREP, a Novel Protein Necessary for Gliding Motility of the Malaria Sporozoite. Int. J. Parasitol. 39, 489–496. doi: 10.1016/j.ijpara.2008.10.004 19000911

[B19] Coppi A. Natarajan R. Pradel G. Bennett B. L. James E. R. Roggero M. A.. (2011). The Malaria Circumsporozoite Protein has Two Functional Domains, Each With Distinct Roles as Sporozoites Journey From Mosquito to Mammalian Host. J. Exp. Med. 208, 341–356. doi: 10.1084/jem.20101488 21262960 PMC3039851

[B20] Coppi A. Pinzon-Ortiz C. Hutter C. Sinnis P. (2005). The *Plasmodium* Circumsporozoite Protein is Proteolytically Processed During Cell Invasion. J. Exp. Med. 201, 27–33. doi: 10.1084/jem.20040989 15630135 PMC1995445

[B21] Coppi A. Tewari R. Bishop J. R. Bennett B. L. Lawrence R. Esko J. D.. (2007). Heparan Sulfate Proteoglycans Provide a Signal to *Plasmodium* Sporozoites to Stop Migrating and Productively Invade Host Cells. Cell Host Microbe 2, 316–327. doi: 10.1016/j.chom.2007.10.002 18005753 PMC2117360

[B22] Costa D. M. Sá M. Teixeira A. R. Loureiro I. Thouvenot C. Golba S.. (2018). TRSP Is Dispensable for the *Plasmodium* Pre-Erythrocytic Phase. Sci. Rep. 8, 15101. doi: 10.1038/s41598-018-33398-8 30305687 PMC6180128

[B23] Cowan G. Krishna S. Crisanti A. Robson K. (1992). Expression of Thrombospondin-Related Anonymous Protein in *Plasmodium falciparum* Sporozoites. Lancet 339, 1412–1413. doi: 10.1016/0140-6736(92)91229-2 1350818

[B24] Cowman A. F. Tonkin C. J. Tham W. H. Duraisingh M. T. (2017). The Molecular Basis of Erythrocyte Invasion by Malaria Parasites. Cell Host Microbe 22, 232–245. doi: 10.1016/j.chom.2017.07.003 28799908 PMC12801281

[B25] Deng M. Templeton T. J. London N. R. Bauer C. Schroeder A. A. Abrahamsen M. S. (2002). *Cryptosporidium parvum* Genes Containing Thrombospondin Type 1 Domains. Infect. Immun. 70, 6987–6995. doi: 10.1128/IAI.70.12.6987-6995.2002 12438378 PMC132954

[B26] Dessens J. T. Beetsma A. L. Dimopoulos G. Wengelnik K. Crisanti A. Kafatos F. C.. (1999). CTRP is Essential for Mosquito Infection by Malaria Ookinetes. EMBO J. 18, 6221–6227. doi: 10.1093/emboj/18.22.6221 10562534 PMC1171685

[B27] Diaz S. A. Martin S. R. Grainger M. Howell S. A. Green J. L. Holder A. A. (2014). *Plasmodium falciparum* Aldolase and the C-Terminal Cytoplasmic Domain of Certain Apical Organellar Proteins Promote Actin Polymerization. Mol. Biochem. Parasitol. 197, 9–14. doi: 10.1016/j.molbiopara.2014.09.006 25261592 PMC4251702

[B28] Diaz S. A. Martin S. R. Howell S. A. Grainger M. Moon R. W. Green J. L.. (2016). The Binding of *Plasmodium falciparum* Adhesins and Erythrocyte Invasion Proteins to Aldolase is Enhanced by Phosphorylation. PloS One 11, 1–20. doi: 10.1371/journal.pone.0161850 PMC5015959 27607074

[B29] Doud M. B. Koksal A. C. Mi L. Z. Song G. Lu C. Springer T. A. (2012). Unexpected Fold in the Circumsporozoite Protein Target of Malaria Vaccines. Proc. Natl. Acad. Sci. U. S. A. 109, 7817–7822. doi: 10.1073/pnas.1205737109 22547819 PMC3356675

[B30] Dundas K. Shears M. J. Sun Y. Hopp C. S. Crosnier C. Metcalf T.. (2018). Alpha-V–Containing Integrins are Host Receptors for the *Plasmodium falciparum* Sporozoite Surface Protein, TRAP. Proc. Natl. Acad. Sci. U. S. A. 115, 4477–4482. doi: 10.1073/pnas.1719660115 29632205 PMC5924908

[B31] Ecker A. Bushell E. S. C. Tewari R. Sinden R. E. (2008). Reverse Genetics Screen Identifies Six Proteins Important for Malaria Development in the Mosquito. Mol. Microbiol. 70, 209–220. doi: 10.1111/j.1365-2958.2008.06407.x 18761621 PMC2658712

[B32] Ejigiri I. Ragheb D. R. T. Pino P. Coppi A. Bennett B. L. Soldati-Favre D.. (2012). Shedding of TRAP by a Rhomboid Protease From the Malaria Sporozoite Surface is Essential for Gliding Motility and Sporozoite Infectivity. PloS Pathog. 8 (7), e1002725. doi: 10.1371/journal.ppat.1002725 22911675 PMC3406075

[B33] Ferguson D. J. P. Balaban A. E. Patzewitz E. M. Wall R. J. Hopp C. S. Poulin B.. (2014). The Repeat Region of the Circumsporozoite Protein is Critical for Sporozoite Formation and Maturation in *Plasmodium* . PloS One 9, 1–25. doi: 10.1371/journal.pone.0113923 PMC4250072 25438048

[B34] Frénal K. Dubremetz J.-F. Lebrun M. Soldati-Favre D. (2017). Gliding Motility Powers Invasion and Egress in Apicomplexa. Nat. Rev. Microbiol. 15, 645–660. doi: 10.1038/nrmicro.2017.86 28867819

[B35] Gaffar F. R. Yatsuda A. P. Franssen F. F. J. J. de Vries E. (2004). A *Babesia bovis* Merozoite Protein With a Domain Architecture Highly Similar to the Thrombospondin-Related Anonymous Protein (TRAP) Present in *Plasmodium* Sporozoites. Mol. Biochem. Parasitol. 136, 25–34. doi: 10.1016/j.molbiopara.2004.02.006 15138064

[B36] Gantt S. Persson C. Rose K. Birkett A. J. Abagyan R. Nussenzweig V. (2000). Antibodies Against Thrombospondin-Related Anonymous Protein do Not Inhibit *Plasmodium* Sporozoite Infectivity *In Vivo.* Infection and Immunity. 68 6), 3667–3673. doi: 10.1128/IAI.68.6.3667-3673.2000 PMC97657 10816526

[B37] Ghosh A. K. Devenport M. Jethwaney D. Kalume D. E. Pandey A. Anderson V. E.. (2009). Malaria Parasite Invasion of the Mosquito Salivary Gland Requires Interaction Between the *Plasmodium* TRAP and the *Anopheles* Saglin Proteins. PloS Pathog. 5 (1), e1000265. doi: 10.1371/journal.ppat.1000265 19148273 PMC2613030

[B38] Goo Y. K. Ueno A. Terkawi M. A. Oluga Aboge G. Junya Y. Igarashi M.. (2013). Actin Polymerization Mediated by *Babesia Gibsoni* Aldolase is Required for Parasite Invasion. Exp. Parasitol. 135, 42–49. doi: 10.1016/j.exppara.2013.06.002 23792005

[B39] Gras S. Jackson A. Woods S. Pall G. Whitelaw J. Leung J. M.. (2017). Parasites Lacking the Micronemal Protein MIC2 are Deficient in Surface Attachment and Host Cell Egress, But Remain Virulent *In Vivo* . Wellcome Open Res. 2, 1–27. doi: 10.12688/wellcomeopenres.11594.1 28630943 PMC5473411

[B40] Gupta R. Mishra A. Choudhary H. H. Narwal S. K. Nayak B. Srivastava P. N.. (2020). Secreted Protein With Altered Thrombospondin Repeat (SPATR) Is Essential for Asexual Blood Stages but not Required for Hepatocyte Invasion by the Malaria Parasite *Plasmodium berghei* . Mol Microbiol. 113, 478–491. doi: 10.1111/mmi.14432 31755154

[B41] Hegge S. Munter S. Steinbüchel M. Heiss K. Engel U. Matuschewski K.. (2010). Multistep Adhesion of *Plasmodium* Sporozoites. FASEB J. 24, 2222–2234. doi: 10.1096/fj.09-148700 20159960

[B42] Hegge S. Uhrig K. Streichfuss M. Kynast-Wolf G. Matuschewski K. Spatz J. P.. (2012). Direct Manipulation of Malaria Parasites With Optical Tweezers Reveals Distinct Functions of *Plasmodium* Surface Proteins. ACS Nano 6, 4648–4662. doi: 10.1021/nn203616u 22568891

[B43] Heintzelman M. B. (2015). Gliding Motility in Apicomplexan Parasites. Semin. Cell Dev. Biol. 46, 135–142. doi: 10.1016/j.semcdb.2015.09.020 26428297

[B44] Heiss K. Nie H. Kumar S. Daly T. M. Bergman L. W. Matuschewski K. (2008). Functional Characterization of a Redundant *Plasmodium* TRAP Family Invasin, TRAP-Like Protein, by Aldolase Binding and a Genetic Complementation Test. Eukaryot. Cell 7, 1062–1070. doi: 10.1128/EC.00089-08 18441124 PMC2446664

[B45] Hellmann J. K. Münter S. Kudryashev M. Schulz S. Heiss K. Müller A. K.. (2011). Environmental Constraints Guide Migration of Malaria Parasites During Transmission. PloS Pathog. 7 (6), e1002080. doi: 10.1371/journal.ppat.1002080 21698220 PMC3116815

[B46] Herrera R. Anderson C. Kumar K. Molina-Cruz A. Nguyen V. Burkhardt M.. (2015). Reversible Conformational Change in the *Plasmodium falciparum* Circumsporozoite Protein Masks its Adhesion Domains. Infect. Immun. 83, 3771–3780. doi: 10.1128/IAI.02676-14 26169272 PMC4567636

[B47] Huynh M. H. Rabenau K. E. Harper J. M. Beatty W. L. Sibley L. D. Carruthers V. B. (2003). Rapid Invasion of Host Cells by *Toxoplasma* Requires Secretion of the MIC2-M2AP Adhesive Protein Complex. EMBO J. 22, 2082–2090. doi: 10.1093/emboj/cdg217 12727875 PMC156089

[B48] Jacot D. Tosetti N. Pires I. Stock J. Graindorge A. Hung Y. F.. (2016). An Apicomplexan Actin-Binding Protein Serves as a Connector and Lipid Sensor to Coordinate Motility and Invasion. Cell Host Microbe 20, 731–743. doi: 10.1016/j.chom.2016.10.020 27978434

[B49] Jalovecka M. Hajdusek O. Sojka D. Kopacek P. Malandrin L. (2018). The Complexity of Piroplasms Life Cycles. Front. Cell. Infect. Microbiol. 8. doi: 10.3389/fcimb.2018.00248 PMC6065256 30083518

[B50] Jalovecka M. Sojka D. Ascencio M. Schnittger L. (2019). *Babesia* Life Cycle – When Phylogeny Meets Biology. Trends Parasitol. 35, 356–368. doi: 10.1016/j.pt.2019.01.007 30733093

[B51] Jewett T. J. Sibley L. D. (2003). Aldolase Forms a Bridge Between Cell Surface Adhesins and the Actin Cytoskeleton in Apicomplexan Parasites. Mol. Cell 11, 885–894. doi: 10.1016/S1097-2765(03)00113-8 12718875

[B52] Kaiser K. Matuschewski K. Camargo N. Ross J. Kappe S. H. I. (2004). Differential Transcriptome Profiling Identifies *Plasmodium* Genes Encoding Pre-Erythrocytic Stage-Specific Proteins. Mol. Microbiol. 51, 1221–1232. doi: 10.1046/j.1365-2958.2003.03909.x 14982620

[B53] Kaneko O. Templeton T. J. Iriko H. Tachibana M. Otsuki H. Takeo S.. (2006). The *Plasmodium* Vivax Homolog of the Ookinete Adhesive Micronemal Protein, CTRP. Parasitol. Int. 55, 227–231. doi: 10.1016/j.parint.2006.04.003 16822707

[B54] Kappe S. Bruderer T. Gantt S. Fujioka H. Nussenzweig V. Ménard R. (1999). Conservation of a Gliding Motility and Cell Invasion Machinery in Apicomplexan Parasites. J. Cell Biol. 147, 937–943. doi: 10.1083/jcb.147.5.937 10579715 PMC2169348

[B55] Keeley A. Soldati D. (2004). The Glideosome: A Molecular Machine Powering Motility and Host-Cell Invasion by Apicomplexa. Trends Cell Biol. 14, 528–532. doi: 10.1016/j.tcb.2004.08.001 15450974

[B56] Kehrer J. Frischknecht F. Mair G. R. (2016). Proteomic Analysis of the *Plasmodium berghei* Gametocyte Egressome and Vesicular Bioid of Osmiophilic Body Proteins Identifies Merozoite Trap-Like Protein (MTRAP) as an Essential Factor for Parasite Transmission. Mol. Cell. Proteomics 15, 2852–2862. doi: 10.1074/mcp.M116.058263 27371728 PMC5013303

[B57] Klug D. Frischknecht F. (2017). Motility Precedes Egress of Malaria Parasites From Oocysts. Elife 6, e19157. doi: 10.7554/eLife.19157 28115054 PMC5262382

[B58] Klug D. Goellner S. Kehrer J. Sattler J. Singer M. Lu C.. (2020). Evolutionarily Distant I Domains can Functionally Replace the Essential Ligand- Binding Domain of *Plasmodium* TRAP. Elife 9, e57572. doi: 10.7554/eLife.57572 32648541 PMC7351488

[B59] Klug D. Kehrer J. Frischknecht F. Singer M. (2018). A Synthetic Promoter for Multi-Stage Expression to Probe Complementary Functions of *Plasmodium* Adhesins. J. Cell Sci. 131 (20), jcs210971. doi: 10.1242/jcs.210971 30237220

[B60] Krause P. J. (2019). Human Babesiosis. Int. J. Parasitol. 49, 165–174. doi: 10.1016/j.ijpara.2018.11.007 30690090

[B61] Labaied M. Camargoa N. Kappe S. H. I. (2007). Depletion of the *Plasmodium berghei* Thrombospondin-Related Sporozoite Protein Reveals a Role in Host Cell Entry by Sporozoites. Mol. Biochem. Parasitol. 153, 159–166. doi: 10.1016/j.molbiopara.2007.03.001 17418435

[B62] Laurens M. B. (2020). RTS,S/AS01 Vaccine (Mosquirix™): An Overview. Hum. Vaccines Immunother. 16, 480–489. doi: 10.1080/21645515.2019.1669415 PMC7227679 31545128

[B63] Lerch A. Koepfli C. Hofmann N. E. Messerli C. Wilcox S. Kattenberg J. H.. (2017). Development of Amplicon Deep Sequencing Markers and Data Analysis Pipeline for Genotyping Multi-Clonal Malaria Infections. BMC Genomics 18, 864. doi: 10.1186/s12864-017-4260-y 29132317 PMC5682641

[B64] Liddington R. C. (2014). Structural Aspects of Integrins. Adv. Exp. Med. Biol. 819, 111–126. doi: 10.1007/978-94-17-9153-3_8 25023171

[B65] Lindner S. E. Swearingen K. E. Harupa A. Vaughan A. M. Sinnis P. Moritz R. L.. (2013). Total and Putative Surface Proteomics of Malaria Parasite Salivary Gland Sporozoites. Mol. Cell. Proteomics 12, 1127–1143. doi: 10.1074/mcp.M112.024505 23325771 PMC3650326

[B66] Li F. Templeton T. J. Popov V. Comer J. E. Tsuboi T. Torii M.. (2004). *Plasmodium* Ookinete-Secreted Proteins Secreted Through a Common Micronemal Pathway are Targets of Blocking Malaria Transmission. J. Biol. Chem. 279, 26635–26644. doi: 10.1074/jbc.M401385200 15069061

[B67] Lopaticki S. Yang A. S. P. John A. Scott N. E. Lingford J. P. O’Neill M. T.. (2017). Protein O-Fucosylation in *Plasmodium falciparum* Ensures Efficient Infection of Mosquito and Vertebrate Hosts. Nat. Commun. 8, 561. doi: 10.1038/s41467-017-00571-y 28916755 PMC5601480

[B68] Lovett J. L. Howe D. K. Sibley L. D. (2000). Molecular Characterization of a Thrombospondin-Related Anonymous Protein Homologue in *Neospora caninum* . Mol. Biochem. Parasitol. 107, 33–43. doi: 10.1016/S0166-6851(99)00228-5 10717300

[B69] Mahajan B. Jani D. Chattopadhyay R. Nagarkatti R. Zheng H. Majam V. (2005). Identification, Cloning, Expression, and Characterization of the Dene for *Plasmodium knowlesi* Surface Protein Containing an Altered Thrombospondin Repeat Domain. Infect. Immun. 73, 5402–5409. doi: 10.1128/IAI.73.9.5402-5409.2005 16113256 PMC1231135

[B70] Martins de Almeida M. E. Santos de Vasconcelos M. G. Monteiro Tarragô A. Morais Mariúba L. A. (2021). Circumsporozoite Surface Protein-Based Malaria Vaccines: A Review. Rev. Inst. Med. Trop. Sao Paulo 63, e11. doi: 10.1590/s1678-9946202163011 33533814 PMC7845937

[B71] Matuschewski K. Nunes A. C. Nussenzweig V. Ménard R. (2002). *Plasmodium* Sporozoite Invasion Into Insect and Mammalian Cells is Directed by the Same Dual Binding System. EMBO J. 21, 1597–1606. doi: 10.1093/emboj/21.7.1597 11927544 PMC125935

[B72] McCormick C. J. Tuckwell D. S. Crisanti A. Humphries M. J. Hollingdale M. R. (1999). Identification of Heparin as a Ligand for the A-Domain of *Plasmodium falciparum* Thrombospondin-Related Adhesion Protein. Mol. Biochem. Parasitol. 100, 111–124. doi: 10.1016/S0166-6851(99)00052-3 10376999

[B73] Mehrizi A. A. Zadeh A. J. Zakeri S. Djadid N. D. (2020). Population Genetic Structure Analysis of Thrombospondin-Related Adhesive Protein (TRAP) as a Vaccine Candidate Antigen in Worldwide *Plasmodium falciparum* Isolates. Infect. Genet. Evol. 80, 104197. doi: 10.1016/j.meegid.2020.104197 31954917

[B74] Ménard R. Sultan A. A. Cortes C. Altszuler R. Van Dijk M. R. Janse C. J.. (1997). Circumsporozoite Protein is Required for Development of Malaria Sporozoites in Mosquitoes. Nature 385, 336–339. doi: 10.1038/385336a0 9002517

[B75] Montenegro V. N. Paoletta M. S. Jaramillo Ortiz J. M. Suarez C. E. Wilkowsky S. E. (2020). Identification and Characterization of a *Babesia bigemina* Thrombospondin-Related Superfamily Member, TRAP-1: A Novel Antigen Involved in Merozoite Invasion. Parasites Vectors 13, 602. doi: 10.1186/s13071-020-04469-5 33261638 PMC7705850

[B76] Morahan B. J. Wang L. Coppel R. L. (2009). No TRAP, No Invasion. Trends Parasitol. 25, 77–84. doi: 10.1016/j.pt.2008.11.004 19101208

[B77] Moreira C. K. Templeton T. J. Lavazec C. Hayward R. E. Hobbs C. V. Kroeze H.. (2008). The *Plasmodium* TRAP/MIC2 Family Member, TRAP-Like Protein (TLP), is Involved in Tissue Traversal by Sporozoites. Cell. Microbiol. 10, 1505–1516. doi: 10.1111/j.1462-5822.2008.01143.x 18346224 PMC3937816

[B78] Mota M. M. Pradel G. Vanderberg J. P. Hafalla J. C. R. Frevert U. Nussenzweig R. S.. (2001). Migration of *Plasmodium* Sporozoites Through Cells Before Infection. Science 291, 141–144. doi: 10.1126/science.291.5501.141 11141568

[B79] Müller H. M. Reckmann I. Hollingdale M. R. Bujard H. Robson K. J. Crisanti A. (1993). Thrombospondin Related Anonymous Protein (TRAP) of *Plasmodium Falciparum* Binds Specifically to Sulfated Glycoconjugates and to HepG2 Hepatoma Cells Suggesting a Role for This Molecule in Sporozoite Invasion of Hepatocytes. EMBO J. 12, 2881–2889. doi: 10.1002/j.1460-2075.1993.tb05950.x 8392935 PMC413541

[B80] Münter S. Sabass B. Selhuber-Unkel C. Kudryashev M. Hegge S. Engel U.. (2009). *Plasmodium* Sporozoite Motility is Modulated by the Turnover of Discrete Adhesion Sites. Cell Host Microbe 6, 551–562. doi: 10.1016/j.chom.2009.11.007 20006843

[B81] Myung J. M. Marshall P. Sinnis P. (2004). The *Plasmodium* Circumsporozoite Protein is Involved in Mosquito Salivary Gland Invasion by Sporozoites. Mol. Biochem. Parasitol. 133, 53–59. doi: 10.1016/j.molbiopara.2003.09.002 14668012

[B82] Naitza S. Spano F. Robson K. J. H. Crisanti A. (1998). The Thrombospondin-Related Protein Family of Apicomplexan Parasites: The Gears of the Cell Invasion Machinery. Parasitol. Today 14, 479–484. doi: 10.1016/S0169-4758(98)01346-5 17040860

[B83] Nemetski S. M. Cardozo T. J. Bosch G. Weltzer R. O’Malley K. Ejigiri I.. (2015). Inhibition by Stabilization: Targeting the *Plasmodium falciparum* Aldolase–TRAP Complex. Malar J. 14, 324. doi: 10.1186/s12936-015-0834-9 26289816 PMC4545932

[B84] Noe A. R. Terry F. E. Schanen B. C. Sassano E. Hindocha P. Phares T. W.. (2021). Bridging Computational Vaccinology and Vaccine Development Through Systematic Identification, Characterization, and Downselection of Conserved and Variable Circumsporozoite Protein CD4 T Cell Epitopes From Diverse *Plasmodium Falciparum* Strains. Front. Immunol. 12. doi: 10.3389/fimmu.2021.689920 PMC8217813 34168657

[B85] Pedroni M. J. Sondgeroth K. S. Gallego-Lopez G. M. Echaide I. Lau A. O. T. (2013). Comparative Transcriptome Analysis of Geographically Distinct Virulent and Attenuated *Babesia bovis* Strains Reveals Similar Gene Expression Changes Through Attenuation. BMC Genomics 14, e763. doi: 10.1186/1471-2164-14-763 PMC3826834 24195453

[B86] Pereira L. M. Candido-Silva J. A. De Vries E. Yatsuda A. P. (2011). A New Thrombospondin-Related Anonymous Protein Homologue in *Neospora caninum* (NcMIC2-Like1). Parasitology 138, 287–297. doi: 10.1017/S0031182010001290 20880420

[B87] Pihlajamaa T. Kajander T. Knuuti J. Horkka K. Sharma A. Permi P. (2013). Structure of *Plasmodium falciparum* TRAP (Thrombospondin-Related Anonymous Protein) A Domain Highlights Distinct Features in Apicomplexan Von Willebrand Factor A Homologues. Biochem. J. 450, 469–476. doi: 10.1042/BJ20121058 23317521

[B88] Putignani L. Possenti A. Cherchi S. Pozio E. Crisanti A. Spano F. (2008). The Thrombospondin-Related Protein CpMIC1 (CpTSP8) Belongs to the Repertoire of Micronemal Proteins of *Cryptosporidium parvum* . Mol. Biochem. Parasitol. 157, 98–101. doi: 10.1016/j.molbiopara.2007.09.004 17981348

[B89] Quadt K. A. Streichfuss M. Moreau C. A. Spatz J. P. Frischknecht F. (2016). Coupling of Retrograde Flow to Force Production During Malaria Parasite Migration. ACS Nano 10, 2091–2102. doi: 10.1021/acsnano.5b06417 26792112

[B90] Ramakrishnan C. Dessens J. T. Armson R. Pinto S. B. Talman A. M. Blagborough A. M.. (2011). Vital Functions of the Malarial Ookinete Protein, CTRP, Reside in the A Domains. Int. J. Parasitol. 41, 1029–1039. doi: 10.1016/j.ijpara.2011.05.007 21729699 PMC4068204

[B91] Riglar D. T. Whitehead L. Cowman A. F. Rogers K. L. Baum J. (2016). Localisation-Based Imaging of Malarial Antigens During Erythrocyte Entry Reaffirms a Role for AMA1 But Not MTRAP in Invasion. J. Cell Sci. 129, 228–242. doi: 10.1242/jcs.177741 26604223 PMC4732298

[B92] Robson K. J. H. Frevert U. Reckmann I. Cowan G. Beier J. Scragg I. G.. (1995). Thrombospondin-Related Adhesive Protein (TRAP) of *Plasmodium falciparum*: Expression During Sporozoite Ontogeny and Binding to Human Hepatocytes. EMBO J. 14, 3883–3894. doi: 10.1002/j.1460-2075.1995.tb00060.x 7664729 PMC394467

[B93] Robson K. J. H. Hall J. R. S. Jennings M. W. Harris T. J. R. Marsh K. Newbold C.. (1988). A Highly Conserved Amino-Acid Sequence in Thrombospondin, Properdin and in Proteins From Sporozoites and Blood Stages of a Human Malaria Parasite. Nature 335, 79–82. doi: 10.1038/335079a0 3045563

[B94] RTS, S. C. T. P (2015). Efficacy and Safety of RTS,S/AS01 Malaria Vaccine With or Without a Booster Dose in Infants and Children in Africa: Final Results of a Phase 3, Individually Randomised, Controlled Trial. Lancet 386, 31–45. doi: 10.1016/S0140-6736(15)60721-8 25913272 PMC5626001

[B95] Sadler J. E. Shelton-Inloes B. B. Sorace J. M. Harlan J. M. Titani K. Davie E. W. (1985). Cloning and Characterization of Two cDNAs Coding for Human Von Willebrand Factor. Proc. Natl. Acad. Sci. U. S. A. 82, 6394–6398. doi: 10.1073/pnas.82.19.6394 2864688 PMC390722

[B96] Schürpf T. Springer T. A. (2011). Regulation of Integrin Affinity on Cell Surfaces. EMBO J. 30, 4712–4727. doi: 10.1038/emboj.2011.333 21946563 PMC3243613

[B97] Shen B. Sibley L. D. (2014). *Toxoplasma* Aldolase is Required for Metabolism But Dispensable for Host-Cell Invasion. Proc. Natl. Acad. Sci. U. S. A. 111, 3567–3572. doi: 10.1073/pnas.1315156111 24550496 PMC3948255

[B98] Siddiqui F. A. Dhawan S. Singh S. Singh B. Gupta P. Pandey A. (2013). *P. falciparum* Rhoptry Protein PfTRAMP and Invasion. Cell Microbiol. 15, 1341–1356. doi: 10.1111/cmi.12118 23387921

[B99] Silvie O. Franetich J. F. Charrin S. Mueller M. S. Siau A. Bodescot M.. (2004). A Role for Apical Membrane Antigen 1 During Invasion of Hepatocytes by *Plasmodium Falciparum* Sporozoites. J. Biol. Chem. 279, 9490–9496. doi: 10.1074/jbc.M311331200 14676185

[B100] Singh A. P. Buscaglia C. A. Wang Q. Levay A. Nussenzweig D. R. Walker J. R.. (2007). *Plasmodium* Circumsporozoite Protein Promotes the Development of the Liver Stages of the Parasite. Cell 131, 492–504. doi: 10.1016/j.cell.2007.09.013 17981117

[B101] Soldati-Favre D. (2008). Molecular Dissection of Host Cell Invasion by the Apicomplexans: The Glideosome. Parasite 15, 197–205. doi: 10.1051/parasite/2008153197 18814681

[B102] Song G. Koksal A. C. Lu C. Springer T. A. (2012). Shape Change in the Receptor for Gliding Motility in *Plasmodium* Sporozoites. Proc. Natl. Acad. Sci. U. S. A. 109, 21420–21425. doi: 10.1073/pnas.1218581109 23236185 PMC3535621

[B103] Spano F. Putignani L. Naitza S. Puri C. Wright S. Crisanti A. (1998). Molecular Cloning and Expression Analysis of a *Cryptosporidium parvum* Gene Encoding a New Member of the Thrombospondin Family. Mol. Biochem. Parasitol. 92, 147–162. doi: 10.1016/S0166-6851(97)00243-0 9574918

[B104] Steinbuechel M. Matuschewski K. (2009). Role for the *Plasmodium* Sporozoite-Specific Transmembrane Protein S6 in Parasite Motility and Efficient Malaria Transmission. Cell. Microbiol. 11, 279–288. doi: 10.1111/j.1462-5822.2008.01252.x 19016774 PMC2688672

[B105] Suarez C. E. Bishop R. P. Alzan H. F. Poole W. A. Cooke B. M. (2017). Advances in the Application of Genetic Manipulation Methods to Apicomplexan Parasites. Int. J. Parasitol. 47, 701–710. doi: 10.1016/j.ijpara.2017.08.002 28893636

[B106] Sultan A. A. Thathy V. Frevert U. Robson K. J. H. Crisanti A. Nussenzweig V.. (1997). TRAP is Necessary for Gliding Motility and Infectivity of *Plasmodium* Sporozoites. Cell 90, 511–522. doi: 10.1016/S0092-8674(00)80511-5 9267031

[B107] Swearingen K. E. Lindner S. E. Shi L. Shears M. J. Harupa A. Hopp C. S.. (2016). Interrogating the *Plasmodium* Sporozoite Surface: Identification of Surface-Exposed Proteins and Demonstration of Glycosylation on CSP and TRAP by Mass Spectrometry-Based Proteomics. PloS Pathog. 12, 1–32. doi: 10.1371/journal.ppat.1005606 PMC4851412 27128092

[B108] Tan K. Duquette M. Liu J. H. Dong Y. Zhang R. Joachimiak A.. (2002). Crystal Structure of the TSP-1 Type 1 Repeats: A Novel Layered Fold and its Biological Implication. J. Cell Biol. 159, 373–382. doi: 10.1083/jcb.200206062 12391027 PMC2173040

[B109] Templeton T. J. Kaslow D. C. Fidock D. A. (2000). Developmental Arrest of the Human Malaria Parasite *Plasmodium falciparum* Within the Mosquito Midgut *via* CTRP Gene Disruption. Mol. Microbiol. 36, 1–9. doi: 10.1046/j.1365-2958.2000.01821.x 10760158

[B110] Terkawi M. A. Ratthanophart J. Salama A. AbouLaila M. Asada M. Ueno A.. (2013). Molecular Characterization of a New *Babesia Bovis* Thrombospondin-Related Anonymous Protein (BbTRAP2). PloS One 8, 2–11. doi: 10.1371/journal.pone.0083305 PMC3862764 24349483

[B111] Tewari R. Spaccapelo R. Bistoni F. Holder A. A. Crisanti A. (2002). Function of Region I and II Adhesive Motifs of *Plasmodium falciparum* Circumsporozoite Protein in Sporozoite Motility and Infectivity. J. Biol. Chem. 277, 47613–47618. doi: 10.1074/jbc.M208453200 12244064

[B112] Thompson J. Cooke R. E. Moore S. Anderson L. F. Janse C. J. Waters A. P. (2004). PTRAMP; a Conserved *Plasmodium* Thrombospondin-Related Apical Merozoite Protein. Mol. Biochem. Parasitol. 134, 225–232. doi: 10.1016/j.molbiopara.2003.12.003 15003842

[B113] Tomley F. M. Billington K. J. Bumstead J. M. Clark J. D. Monaghan P. (2001). EtMIC4: A Microneme Protein From *Eimeria tenella* That Contains Tandem Arrays of Epidermal Growth Factor-Like Repeats and Thrombospondin Type-I Repeats. Int. J. Parasitol. 31, 1303–1310. doi: 10.1016/S0020-7519(01)00255-7 11566298

[B114] Tossavainen H. Pihlajamaa T. Huttunen T. K. Raulo E. Rauvala H. Permi P.. (2006). The Layered Fold of the TSR Domain of *P. falciparum* TRAP Contains a Heparin Binding Site. Protein Sci. 15, 1760–1768. doi: 10.1110/ps.052068506 16815922 PMC2242559

[B115] Trottein F. Triglia T. Cowman A. F. (1995). Molecular Cloning of a Gene From *Plasmodium falciparum* That Codes for a Protein Sharing Motifs Found in Adhesive Molecules From Mammals and Plasmodia. Mol. Biochem. Parasitol. 74, 129–141. doi: 10.1016/0166-6851(95)02489-1 8719155

[B116] Tucker R. P. (2004). The Thrombospondin Type 1 Repeat Superfamily. Int. J. Biochem. Cell Biol. 36, 969–974. doi: 10.1016/j.biocel.2003.12.011 15094110

[B117] Uchime O. Herrera R. Reiter K. Kotova S. Shimp R. L. Miura K.. (2012). Analysis of the Conformation and Function of the *Plasmodium falciparum* Merozoite Proteins MTRAP and PTRAMP. Eukaryot. Cell 11, 615–625. doi: 10.1128/EC.00039-12 22467743 PMC3346429

[B118] Ueti M. W. Johnson W. C. Kappmeyer L. S. Herndon D. R. Mousel M. R. Reif K. E.. (2020). Comparative Analysis of Gene Expression Between *Babesia bovis* Blood-Stages and Kinetes Allowed Improved Genome Annotation. Int. J. Parasitol. 51 (2-3), 123–136. doi: 10.1016/j.ijpara.2020.08.006 33069745

[B119] Venugopal K. Hentzschel F. Valkiūnas G. Marti M. (2020). *Plasmodium* Asexual Growth and Sexual Development in the Haematopoietic Niche of the Host. Nat. Rev. Microbiol. 18, 177–189. doi: 10.1038/s41579-019-0306-2 31919479 PMC7223625

[B120] Wang Q. Fujioka H. Nussenzweig V. (2005). Exit of *Plasmodium* Sporozoites From Oocysts is an Active Process That Involves the Circumsporozoite Protein. PloS Pathog. 1, 0072–0079. doi: 10.1371/journal.ppat.0010009 PMC1238744 16201021

[B121] Wengelnik K. Spaccapelo R. Naitza S. Robson K. J. H. Janse C. J. Bistoni F.. (1999). The A-Domain and the Thrombospondin-Related Motif of *Plasmodium falciparum* TRAP are Implicated in the Invasion Process of Mosquito Salivary Glands. EMBO J. 18, 5195–5204. doi: 10.1093/emboj/18.19.5195 10508153 PMC1171590

[B122] Whittaker C. A. Hynes R. O. (2002). Distribution and Evolution of Von Willebrand/Integrin A Domains: Widely Dispersed Domains With Roles in Cell Adhesion and Elsewhere. Mol. Biol. Cell 13, 3369–3387. doi: 10.1091/mbc.E02 12388743 PMC129952

[B123] World Health Organization (2021). World Malaria Report 2021. Geneva: World Health Organization.

[B124] Yuda M. Sakaida H. Chinzei Y. (1999). Targeted Disruption of the *Plasmodium berghei* CTRP Gene Reveals its Essential Role in Malaria Infection of the Vector Mosquito. J. Eukaryot. Microbiol. 190, 1711–1715. doi: 10.1084/jem.190.11.1711 PMC2195737 10587361

[B125] Yu L. Liu Q. Zhan X. Huang Y. Sun Y. Nie Z.. (2018). Identification and Molecular Characterization of a Novel *Babesia orientalis* Thrombospondin-Related Anonymous Protein (BoTRAP1). Parasites Vectors 11, 1–9. doi: 10.1186/s13071-018-3245-2 30587207 PMC6307320

[B126] Zhan X. He J. Yu L. Liu Q. Sun Y. Nie Z.. (2019). Identification of a Novel Thrombospondin-Related Anonymous Protein (BoTRAP2) From *Babesia Orientalis* . Parasites Vectors 12, 1–8. doi: 10.1186/s13071-019-3457-0 31053087 PMC6500065

[B127] Zhou J. Fukumoto S. Jia H. Yokoyama N. Zhang G. Fujisaki K.. (2006). Characterization of the *Babesia gibsoni* P18 as a Homologue of Thrombospondin Related Adhesive Protein. Mol. Biochem. Parasitol. 148, 190–198. doi: 10.1016/j.molbiopara.2006.03.015 16675041

